# A portable nanobiosensor with machine learning: enabling multi-cytokines point-of-care testing and early identification of sepsis immunoparalysis

**DOI:** 10.1186/s12951-026-04939-5

**Published:** 2026-08-14

**Authors:** Rongjun Yu, Jiangling Wu, Anyi Chen, Jianjiang Xue, Sihan Guo, Siling Chen, Shanshan Dong, Li Li, Xiao Gui, LiLin Wang, Gang Tian, Gang Liu, Jingfu Qiu, Weixian Chen

**Affiliations:** 1 Department of Clinical Laboratory and Medical Sciences Research Center, University-Town Hospital of Chongqing Medical University, Chongqing, 401331 P. R. China; 2 School of Public Health and Management, Chongqing Medical University, Chongqing, 400016 P. R. China; 3 Department of Laboratory Medicine, The Second Affiliated Hospital of Chongqing Medical University, Chongqing, 400016 P. R. China; 4 Department of Emergency, Yongchuan Hospital of Chongqing Medical University, Chongqing, 402160 P. R. China; 5 Department of Laboratory Medicine, The Affiliated Hospital of Southwest Medical University, Sichuan Province Engineering Technology Research Center of Molecular Diagnosis of Clinical Diseases, Molecular Diagnosis of Clinical Diseases Key Laboratory of Luzhou, Sichuan, 646000 P. R. China

**Keywords:** Nanozyme-linked immunosorbent assay, Portable nanobiosensor, Machine learning, Multi-cytokines point-of-care testing, Sepsis immunoparalysis

## Abstract

Sepsis immunoparalysis (SIs) is a major factor contributing to organ dysfunction. However, emergency departments (EDs) and intensive care units (ICUs) currently lack a rapid workflow for early identification of SIs. Herein, an innovative strategy for early SIs identification is proposed. We construct a portable nanobiosensor based on the Nanozyme-Linked Immunosorbent Assay (NLISA) for ultrasensitive and specific Multi-Cytokines quantification, benefiting from its advantages of parallel, independent, rapid, and portable detection. Machine learning is applied to process Multi-Cytokines concentration data measured under varied conditions and build an early identification model for rapid and effective sepsis immunoparalysis diagnosis. This method achieves point-of-care testing (POCT) with small plasma sample volumes (30 µL), with a detection limit as low as 3.8 fg mL^− 1^. Notably, machine learning models constructed from different cytokine combinations can achieve high diagnostic performance, accurately distinguishing sepsis immunoparalysis, sepsis non-immunoparalysis, and healthy donors, while addressing diverse clinical needs and cost-effectiveness considerations, enabling personalized diagnostic strategies tailored to distinct patient populations and resource-constrained settings. This opens up the potential application of the nanobiosensor with machine learning for POCT of Multi-Cytokines detection and sepsis immunoparalysis early identification.

**Supplementary Information:**

The online version contains supplementary material available at https://doi.org/10.1186/s12951-026-04939-5.

## Introduction

Sepsis is characterized as a life-threatening organ dysfunction due to dysregulated host response to infection based on the definition of the Third International Consensus Definitions for Sepsis and Septic Shock (Sepsis-3.0) [1, 2]. It is a biphasic disease characterized by an initial inflammatory phase (shock) followed by a prolonged immunoparalysis phase [3–6]. While shock accounts for about 15% of sepsis-related mortality, the immunoparalysis phase accounts for 85% of deaths, usually due to the state of immunoparalysis leading to secondary double infections and multiple organ dysfunction syndrome (MODS) [7–10]. Nevertheless, there remains no definitive clinical timing window for diagnosing sepsis immunoparalysis. Clinicians currently judge immunoparalysis retrospectively based on treatment response, which prevents timely determination of the optimal window for initiating targeted immunotherapy and often delays intervention by missing the critical “golden hour” [11–13]. This underscores the urgent clinical need to find potentially effective biomarkers and develop efficient detection approaches to achieve early identification of sepsis immunoparalysis [14–16].

Cytokines are small-molecule proteins (molecular weight < 40 KDa) secreted by cells [17]. They exert diverse biological activities, mediate intercellular signal transduction and immune regulation, and act as sensitive biomarkers to evaluate sepsis severity and predict patient mortality [18]. Massive cytokine release occurs upon sepsis onset to modulate systemic immune and inflammatory responses. During sepsis, pathogenic microorganisms and the toxins they release trigger the release of a large number of inflammatory cytokines through a “cascade-like” reaction, leading to a systemic inflammatory response syndrome (SIRS) [19–21]. Subsequently, the continuous and massive release of inflammatory cytokines leads to the compensatory synthesis and secretion of anti-inflammatory cytokines, thereby attenuating the body’s inflammatory response. However, the continuous and excessive production of anti-inflammatory cytokines drives compensatory anti-inflammatory response syndrome (CARS), placing the body in an " immunoparalysis state " [22–24]. Therefore, numerous current studies emphasize the crucial role of dynamically monitoring inflammatory and anti-inflammatory cytokines in the early identification of immunoparalysis in sepsis [25–29]. Consistent with these findings, our data reveal that significant dynamic changes in cytokine levels do occur in some sepsis patients with immunoparalysis, specifically characterized by a decrease in inflammatory cytokines levels and an increase in anti-inflammatory cytokines levels. These findings collectively support the notion that Multi-Cytokines can serve as potential objective biomarkers for the early identification of sepsis immunoparalysis.

However, given the diverse range and low abundance of Multi-Cytokines in the bodies of patients with early-stage sepsis immunoparalysis, the sensitivity and detection range of traditional Enzyme-Linked Immunosorbent Assay (ELISA) kits fall significantly short of meeting the clinical demand [30, 31]. Although Chemiluminescence (CL) [32, 33] and Flow cytometry (FCM) [34] exhibit higher sensitivity compared to ELISA, considering the rapid progression and complex dynamics of sepsis, these two methods suffer from cumbersome operational procedures and high technical requirements for laboratory personnel, resulting in slow detection speeds that are inadequate for meeting the timeliness needs of point-of-care testing (POCT) in outpatient and bedside settings. Furthermore, existing biosensors and cytokine detection platforms are generally limited by low sensitivity, inability to achieve multiplexed detection, and dependence on complex instrumentation and trained personnel, which further restricts their clinical translatability [35–39]. Importantly, due to factors such as insufficient sample sizes and the lack of longitudinal studies, constructing an effective early identification model for early-stage immunoparalysis poses numerous challenges. Therefore, there is an urgent need to develop an efficient and reliable Multi-Cytokines detection method to establish a model suitable for the early identification of sepsis immunoparalysis [40]. This would enable the rapid identification of immunoparalysis states and the timely implementation of immune intervention measures to restore the homeostatic balance of the body’s inflammatory response.

Machine learning, as an artificial intelligence technology widely applied in the biological field, demonstrates unique advantages in the automated analysis of complex data, the identification of biomarkers, and the construction of precision medicine early identification models [41–43]. Therefore, in this work, we introduce a portable nanobiosensor based on Nanozyme-Linked Immunosorbent Assay (NLISA) for POCT of Multi-Cytokines at the bedside. By combining this technology with machine learning, we have constructed an early identification model for sepsis immunoparalysis. The portable nanobiosensor, featuring a wireless USB-like configuration, offered unparalleled compactness and convenience, enabling direct data transmission via Bluetooth to smartphones or tablets and then the data could be uploaded to a cloud database utilizing an internet connection. The entire system does not require sophisticated device package and fabrication, which underscores its potential for enabling on-site and real-time detection. NLISA serves as a signal catalyst of reporter cytokines detection, and it is a stepwise addition of reagents onto an immobilized surface that ends with the catalysis of a substrate to generate a readout signal. The substrate conversion rate of nanozymes, which are comprised of nanometer-sized catalytic metallic particles, is higher than their enzymatic counterparts, thus making NLISA even more sensitive than ELISA. Furthermore, in a previous study, we have demonstrated that when the Ti_3_C_2_Tx MXene nanozyme is combined with alkaline phosphatase in a 1-naphthyl phosphate (1-NPP) substrate solution, a novel cascade catalytic amplification strategy is generated, enabling efficient electrochemical signal amplification [44]. Moreover, by utilizing machine learning algorithms in conjunction with NLISA, we were able to analyze Multi-Cytokines with high accuracy to identify sepsis immunoparalysis (SIs), sepsis non-immunoparalysis (SNIs) and healthy donors (HDs) in both training and validation cohorts. Notably, by constructing multiple machine learning models tailored to different cytokine combinations, this platform enables further personalized detection. Therefore, we believe that the portable nanobiosensor provides a promising tool for POCT of Multi-Cytokines. Coupled with machine learning for constructing an early identification model, it opens up an entirely new technological platform for early identification of sepsis immunoparalysis.

## Experimental section

### Experimental design

The objective of our study was to establish a robust and portable nanobiosensor using NLISA platform for POCT of Multi-Cytokines and integrate machine learning to construct an early identification model for sepsis immunoparalysis. The nanobiosensor was designed to enable the rapid detection of a panel of cytokine biomarkers using minimal sample fluid (30 µL) in human blood plasma. Sensor performance metrics (sensitivity, specificity, dynamic range, detection limit, precision, and accuracy) were tested for Interleukin-6 (IL-6), IL-1β, IL-4, IL-10, Tumor Necrosis Factor‑α (TNF-α), and Transforming Growth Factor‑β (TGF-β) in plasma. The nanobiosensor was further validated for clinical translation using machine learning, accurately analyzing Multi-Cytokines in SIs, SNIs, and HDs cohorts, demonstrating its robustness and reliability.

### Reagents and materials

Ti_3_C_2_T_x_ (MXene) few layer dispersion solution (Lateral size 2–5 μm) was obtained from Jiangsu XFNANO Materials Tech. Co., Ltd. (Nanjing, China). Nafion solutions were obtained from Aladdin Biochemical Tech. Co., Ltd. (Shanghai, China). Gold chloride (HAuCl_4_∙4H_2_O), sodium citrate, bovine serum albumin (BSA), 1-naphthyl phosphate (1-NPP), 1-naphthol and diethanolamine (DEA) were purchased from Sigma-Aldrich Chemical (St. Louis, USA). IL-6, IL-1β, TNF-α, IL-4, IL-10, TGF-β, their respective antibodies including anti-IL-6, anti-IL-1β, anti-TNF-α, anti-IL-4, anti-IL-10, anti-TGF-β, as well as the secondary antibodies against these antigens labeled with alkaline phosphatase (AP-Ab2), are all provided by Beijing Bioss Biotechnology Co., Ltd. (Beijing, China). Magnetic microspheres coated with specific cytokine antibodies, AP-labeled specific cytokine antibodies, and TGF-β ELISA kits were purchased from Beijing Hotgen (Beijing, China). PE anti-human HLA-DR and PerCP/Cyanine 5.5 anti-human CD14 were obtained from BioLegend (SanDiego, USA). Red Blood Cell Lysis Buffer was purchased from Solarbio (Beijing, China). Clinical plasma samples were obtained from the University-Town Hospital of Chongqing Medical University (Chongqing, China). All the stock proteins and patient samples were stored at −80 ℃ or according to their storage conditions until further use. None of the proteins underwent more than 3 freeze-thaw cycles to avoid denaturing of the proteins. The antibodies were diluted in PBS to bring them to their optimized concentration, while their respective antigens were spiked in pooled human plasma in varying concentrations to perform calibrated response curves for each target biomarker.

The buffers and apparatus involved in this experiment are displayed in Supporting Material.

### Fabrication of the portable nanobiosensor

Ti_3_C_2_T_x_ MXene/Au NPs-Modified nanobiosensor was prepared to explore the sensing performance and further be used for in situ detection of the Multi-Cytokines of sepsis immunoparalysis. The nanobiosensor utilizes the BIOSYS smart biosensor system platform, which is comprised of the following components: (1) A USB-like portable electrochemical workstation with individually configured electrodes that can detect multiple biomarkers in samples in real-time, ensuring complete independence and mutual non-interference; (2) A tablet equipped with nanobiosensors, which can connect to the electrochemical workstation via Bluetooth for wireless communication, converting biological signals generated by sample reactions into electrical signals for output. The detection signals can be promptly displayed and uploaded to the cloud for subsequent data analysis and processing.

### Construction of POCT based on NLISA

The construction process of the NLISA sandwich-type electrochemical nanobiosensor is outlined as follows. Firstly, Ti_3_C_2_T_x_ MXene (0.1 mg mL^− 1^) was suspended in ultrapure water containing a 0.1% Nafion solution and sonicated for 60 min. Gold nanoparticles (Au NPs) were synthesized according to a typical method, and the detailed procedure was elaborated in Supporting Material. The products were stored at 4 ℃ and protected from light for further use. Subsequently, the Ti_3_C_2_T_x_ MXene/Au NPs (0.1 mg mL^− 1^) was coated on the glassy carbon electrode (GCE) surface polished with alumina slurry (300 and 50 nm) and dried at 60 ℃ for 15 min. Next, 8 µL of Ab1 was immobilized on the modified electrode via Au–N bonds and stored at 4 ℃ overnight. After the incubation period, the excess Ab1 on the electrode surface was washed with PBS solution. To block nonspecific active sites, 8 µL of BSA solution (1 wt%) was added to the modified electrode surface for 25 min at 37 ℃. Then, 5 µL of a series of cytokine standard solutions of different concentrations were incubated on the electrodes at 37 ℃. After that, the AP-Ab2 was added to the electrode surface and incubated to bind the corresponding antigen. Finally, the electrochemical signal was measured in the DEA buffer containing 1.0 mg mL^− 1^ 1-NPP by DPV after rinsing with DEA buffer to remove the unbound AP-Ab2. All parameter configurations of electrochemical measurements are shown in Supporting Material.

### Chemiluminescence assays

The detections of IL-6, IL-1β, IL-4, IL-10, and TNF-α were accomplished using a fully automated immunoassay analyzer. Briefly, the process began by mixing magnetic microspheres coated with specific cytokine antibodies, AP-labeled specific cytokine antibodies, and the sample to be tested, with continuous agitation for 1 h. The cytokines present in the sample bound to these specific antibodies, forming a unique immune complex. Subsequently, unbound, enzyme-labeled antibodies were washed away. Then, a chemiluminescent substrate was added to the immune complex. In the fully automated chemiluminescent immunoassay analyzer, these substrates reacted with the enzymes within the complex, generating a luminescent signal. Based on the correlation between the signal and the cytokine concentration, the analyzer calculated the precise concentration of the cytokines in the sample.

### ELISA assays

The detection of TGF-β was performed using ELISA, following the manufacturer’s instructions. Briefly, 100 µL of plasma samples were pipetted onto an ELISA plate and incubated at 37 °C for 1 h. Subsequently, 100 µL of biotinylated antibodies were added and incubated at 37 °C for another 1 h. After that, the unbound antibodies were washed away, and streptavidin-labeled horseradish peroxidase (HRP) was introduced, followed by incubation at room temperature for 30 min. Once the excess HRP was washed off, 3,3′,5,5′-tetramethylbenzidine (TMB) substrates were added and incubated in the dark for 15 min. Finally, the reaction was terminated with stop solution, and the absorbance of each well was promptly measured at 450 nm using a NanoDrop2000 microplate reader.

### Flow cytometry analysis

Monocyte human leukocyte antigen‑DR (HLA‑DR) expression was quantified via flow cytometry. Briefly, 100 µL EDTA‑K₂-anticoagulated whole blood was incubated with 5 µL PE anti‑human HLA‑DR and 5 µL PerCP/Cyanine 5.5 anti‑human CD14 antibodies. After brief vortexing, samples were incubated on ice away from light for 30 min. Subsequently, 300 µL red blood cell lysis buffer was added, followed by thorough mixing and incubation at room temperature for 2 min. Cells were centrifuged at 350 × g for 10 min; the supernatant was aspirated, and the cell pellet was washed once with physiological saline. Finally, cell pellets were resuspended in 500 µL physiological saline prior to flow cytometric measurement.

### Clinical sample acquisition and assays

Clinical blood samples were obtained from patients with SIs, SNIs, and HDs conditions at the University-Town Hospital of Chongqing Medical University, with prior approval from the hospital’s medical ethics committee. All ethical guidelines and regulations were strictly adhered to. For this study, plasma samples collected from patients with SIs (*n* = 120), SNIs (*n* = 120), and HDs (*n* = 120), within 24 h post-enrollment, were utilized. Specific inclusion and exclusion criteria for patient selection are detailed in Supporting Material. Demographic characteristics, including age, gender, and diagnosis, are summarized in Supporting Table S8. Plasma was separated from blood samples by centrifugation at 3000 rpm for 30 min and stored at −80 °C for subsequent analysis. Subsequently, 5 µL of each plasma sample was incubated in the NLISA system for 1 h at 37 °C. The entire procedure, from blood collection through sample pre-processing to result generation, was completed within 3 h.

### Statistical analysis

All tests were performed at least three times. The mean, standard deviation, and limit of detection (LOD) were calculated using standard formulas. Statistical significance was assessed using the two-tailed Mann-Whitney U test with Bonferroni correction (*p* < 0.05 was considered statistically significant). Receiver operating characteristic (ROC) analyses were performed for Multi-Cytokines signatures to evaluate the area under the curve (AUC), sensitivity, specificity, and accuracy of SIs, SNIs, and HDs diagnosis. The optimal cutoff points were selected using Youden’s index. Figures were prepared using GraphPad Prism (version 9.5, GraphPad Software). Machine learning modeling, statistical analysis, and visualization were performed using Python 3.14.0. Data processing was conducted with pandas and NumPy. Statistical tests were performed using SciPy. Data normalization, stratified train-test splitting, cross-validation, ROC/AUC analysis, confusion matrices, and machine learning models were implemented using scikit-learn. Figures were generated using Matplotlib and Seaborn. Model interpretability was assessed using SHapley Additive exPlanations (SHAP).

## Results and discussion

### Working principle of portable nanobiosensor

**Scheme 1:**
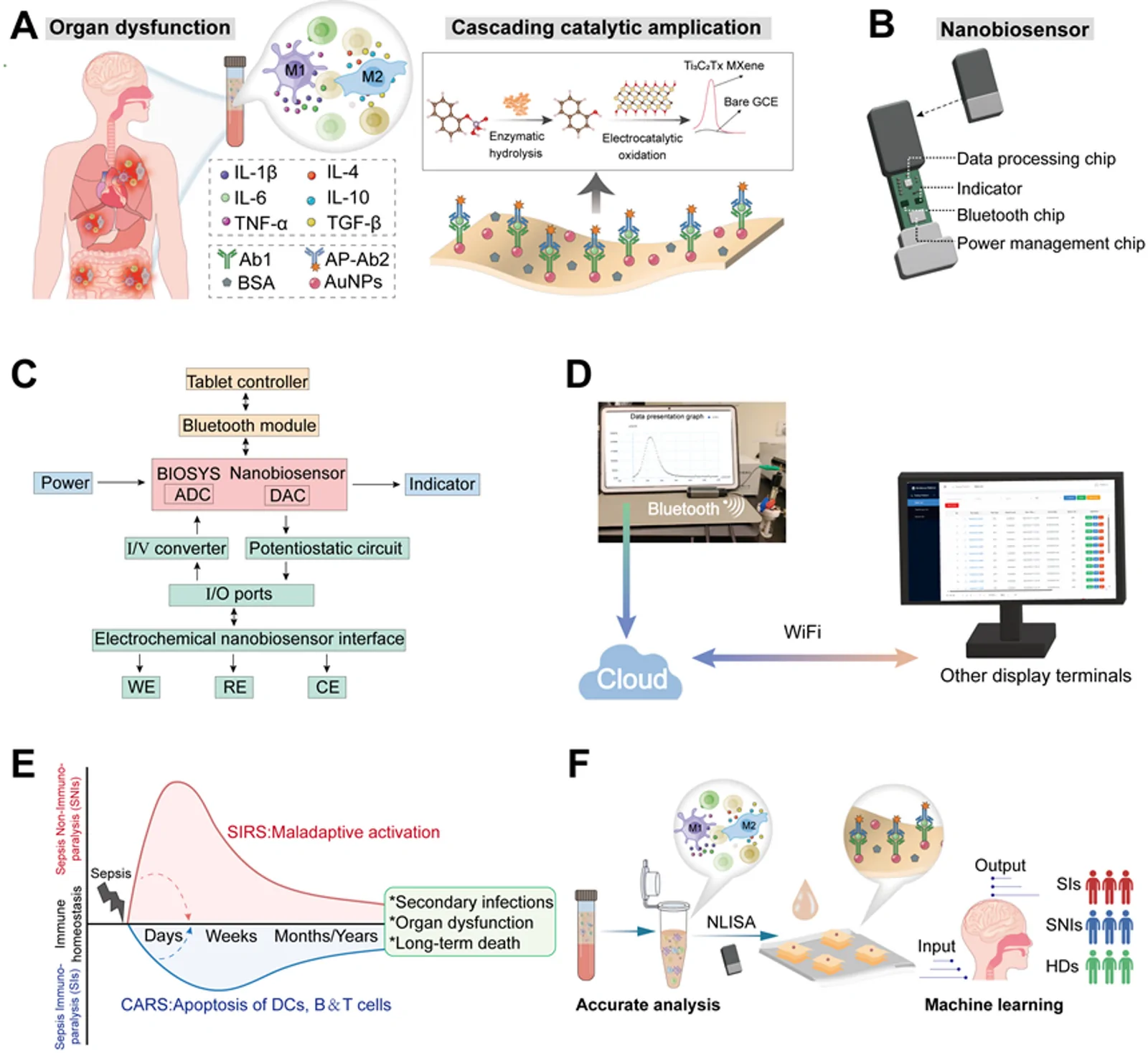
A portable nanobiosensor based on NLISA for POCT of multi-cytokines with machine learning to construct an early identification model for sepsis immunoparalysis. (**A**) The construction of Ti_3_C_2_Tx MXene Nanozyme-Linked Immunosorbent Assay (NLISA). (**B**) The USB-like wireless electrochemical workstation. (**C**) The internal structure and operational mechanism of a USB-like wireless electrochemical workstation. (**D**) The BIOSYS smart biosensor system platform: Biological signals from the NLISA process are converted into electrical signals, which are then displayed in real-time on a tablet via Bluetooth and subsequently uploaded to a cloud database using an internet connection. (**E**) Immune dysregulation in sepsis. (**F**) Sensing signals from samples processed using machine learning to discriminate SIs, SNIs, and HDs

The design of the portable nanobiosensor was based on NLISA assay. As illustrated in Scheme 1A, at the working electrode interface, the cytokine Ab1 is immobilized on a Ti_3_C_2_Tx MXene/Au NPs-modified surface. Upon capturing the corresponding cytokine, an alkaline phosphatase (AP)-conjugated sandwich-type immune complex is formed for current transduction. Our previous research has found that Ti_3_C_2_Tx MXene is an efficient nanozyme with area-dependent electrocatalytic activity in the oxidation of phenolic compounds, which can apparently further improve the sensor’s performance. By combining Ti_3_C_2_Tx MXene with AP, with the addition of 1-NPP in the electrolyte solution, 1-naphthol is produced through AP-catalyzed hydrolysis of 1-NPP. Subsequently, a novel cascade catalytic amplification strategy is constructed through the electrochemical oxidation catalyzed by Ti_3_C_2_Tx MXene, generating amplified electrochemical signals.

The BIOSYS smart biosensor system platform comprises a working electrode detection station, a USB-like radio electrochemical workstation, and a tablet control terminal for parameter setting, detection, observation, and wireless data transmission as illustrated in Scheme 1D. Essentially, the working electrode is interconnected with the microelectrochemical workstation, while the tablet communicates with the electrochemical platform via Bluetooth to control parameters for executing electrochemical detections. The tablet further displays and stores the detection data, with the capability to upload the information to the cloud for sharing. The USB-like radio electrochemical workstation, measuring 45 mm ×21 mm ×12 mm, is a miniature station, with its internal structure depicted in Scheme 1B. Based on a high-precision signal processing sensor, this workstation functions as an electrochemical detector, boasting a current detection range from 10 pA to 500 µA. It is integrated with a rechargeable power source and indicator lights. As shown in Scheme 1C, the signal processing section comprises a processing workstation, digital-to-analog converters (DAC), and analog-to-digital converters (ADC), collectively undertaking the core functionalities. The signal conditioning section, encompassing current-to-voltage converters, constant voltage circuits, and I/O ports, ensures the stability of both input and output signals. Additionally, a Bluetooth module with a maximum communication range of 100 m serves as the signal conduit, facilitating communication between the workstation and the tablet.

Furthermore, the data detected by the portable nanobiosensor has been tightly integrated with machine learning techniques, as illustrated in Scheme 1E and 1F. Leveraging the powerful capabilities of machine learning algorithms, precise analysis, systematic training, and rigorous validation were conducted on the dataset, resulting in high-accuracy classification and early identification predictions for patient cohorts with SIs, SNIs, and HDs.

### Identification of multi-cytokines biomarkers associated with sepsis immunoparalysis

**Fig. 1:**
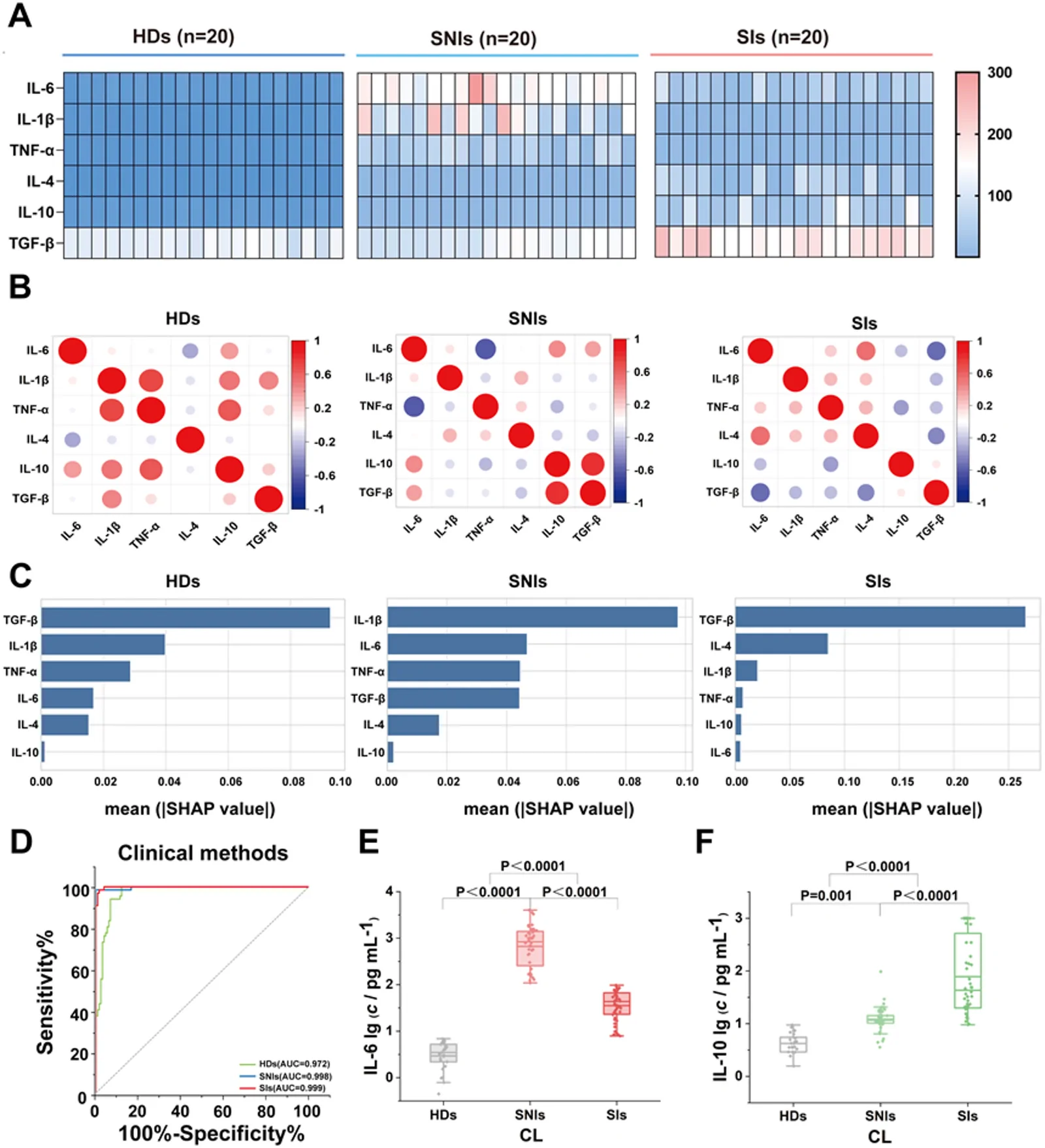
(**A**) Heatmap of unsupervised hierarchical clustering for expression levels of six cytokines across different patient populations. (**B**) Correlation matrices illustrate the expression profiles of the six cytokines in various patient populations. (**C**) SHAP feature importance bar plot showing the mean absolute SHAP value of each cytokine. (**D**) ROC curves and AUC values of clinical methods in differentiating between SIs, SNIs, and HDs. (**E**-**F**) Significant differences in the expression of IL-6 and IL-10 cytokines among SIs, SNIs, and HDs

Multi-Cytokines were determined as potential biomarkers for early identification of sepsis immunoparalysis according to the following criteria: (1) Previous studies have shown that in the negative feedback regulation process of sepsis immunoparalysis, the number and function of various cell components mediated by inflammatory cytokines of the innate immune system (such as IL-6, IL-1β and TNF-α) are downregulated [45–47]. Meanwhile, anti-inflammatory cytokines (including IL-4, IL-10 and TGF-β) are gradually upregulated, which is closely related to the occurrence of immunoparalysis [48–50]. (2) To evaluate the clinical application performance of Multi-Cytokines in the early identification of SIs, we collected 60 plasma samples (Supporting Table S8) involving SIs (*n* = 20), SNIs (*n* = 20) and HDs (*n* = 20). Using these samples, we performed multiparametric profiling with clinical traditional methods like CL and ELISA assays. By unsupervised hierarchical clustering analysis, we investigated whether a panel of six cytokines for each patient exhibited mutually exclusive or similar expression patterns in different patient populations. The expression heatmap showed that the abundance of each cytokine has considerable heterogeneity in different patient populations (Fig. 1A). Specifically, inflammatory cytokines (IL-6, IL-1β and TNF-α) were upregulated in SNIs compared to SIs and HDs plasma samples, whereas anti-inflammatory cytokines (IL-4, IL-10 and TGF-β) were upregulated in SIs compared to SNIs and HDs plasma samples. These comparative analyses revealed the heterogeneity among patient populations and corroborated the validity of the analytical data obtained by CL and ELISA. Subsequently, pairwise comparisons of six cytokines in different patient populations were shown in Fig. 1B. Notably, the six cytokines did not correlate strongly with each other in these patient populations, which drives us to the combinations of multiple markers for the accurate early identification of SIs.

To further improve the clinical identification performance of Multi-Cytokines in differentiating SIs, SNIs, and HDs, we harnessed logistic regression to integrate all cytokine profiles. It should be noted that this logistic regression model does not perform a simple summation of raw cytokine values; instead, it first applies normalization to the input data, thereby eliminating the influence of differing absolute magnitudes across individual cytokine measurements. This ensures that each cytokine contributes to the model on a comparable scale. ROC analyses were then applied to assess the sensitivity, specificity, and accuracy of the Multi-Cytokines assay. As illustrated in Fig. 1D, the Multi-Cytokines assay demonstrated excellent diagnostic performance, with AUC values of 0.999 for SIs, 0.998 for SNIs, and 0.972 for HDs, respectively. To further interpret the biological contributions of individual cytokines to the model output, we performed SHAP analysis, which revealed the relative contribution of each cytokine to the classification signal (Fig. 1C). Figure 1E and F highlight significant differences in IL-6 and IL-10 expression among these groups (the significant differences in the expression of the remaining cytokines are illustrated in Fig. S2). Statistical significance was assessed using the two-tailed Mann-Whitney U test with Bonferroni correction. These results suggest that the changing trends of inflammatory and anti-inflammatory cytokines could potentially serve as biomarkers for early identification of sepsis immunoparalysis. Given the rapid progression of sepsis, dynamic monitoring of Multi-Cytokines changes is necessary.

### Characterization of the portable nanobiosensor

**Fig. 2:**
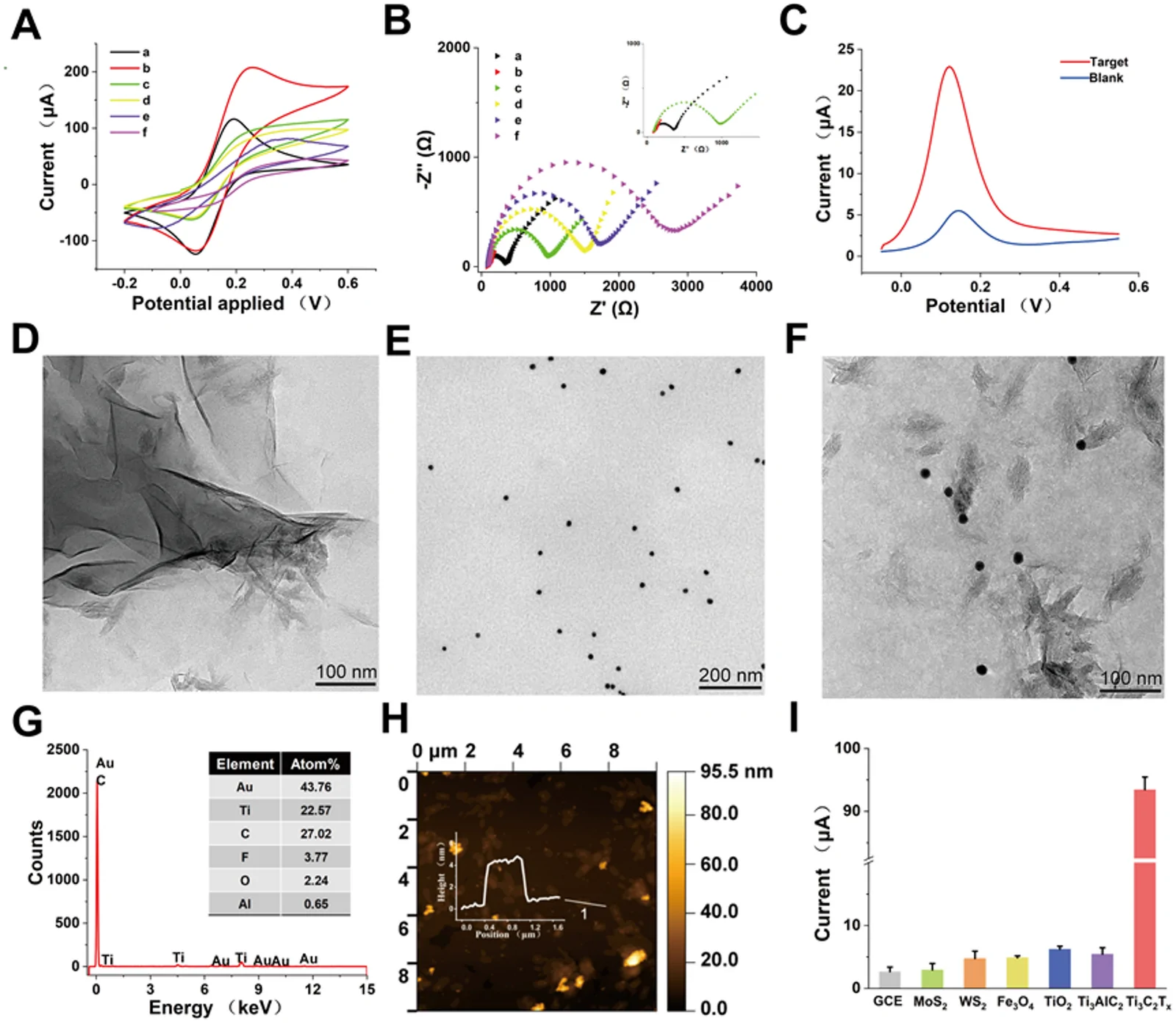
Stepwise fabrication of the working electrode shown by the measurements of (**A**) CV and (**B**) EIS at a bare electrode (**a**), Ti_3_C_2_Tx MXene/Au NPs-modified electrode (**b**), IL-6 Ab1-modified electrode (**c**), BSA-modified electrode (d), IL-6-modified electrode (**e**) and AP-Ab2-modified electrode (**f**). Insert: Amplified Nyquist plot of bare electrode, Ti_3_C_2_Tx MXene/Au NPs-modified electrode and IL-6 Ab1-modified electrode. (**C**) DPV response of a modified Ti_3_C_2_Tx MXene/Au NPs-based sensor before and after incubation with 10 ng mL^− 1^ of IL-6. (**D**-**F**) TEM images of Ti_3_C_2_Tx MXene, Au NPs and Ti_3_C_2_Tx MXene/Au NPs, respectively. (**G**) EDX spectrum and elemental composition of the Ti_3_C_2_Tx MXene/Au NPs composite. (**H**) AFM image of Ti_3_C_2_T_x_ MXene flakes. (**I**) Oxidation current of 1-naphthol measured with different materials modified electrodes. Error bars indicate mean ± standard deviation (s.d.)

Cyclic voltammetry (CV) and electrochemical impedance spectroscopy (EIS) measurements were employed to characterize each stage of nanobiosensor fabrication (Fig. 2A and B). After modification with Ti_3_C_2_Tx MXene/Au NPs, a notable increase in peak current in CV and a decrease in resistance in the Nyquist plot were observed, indicating enhanced electrode conductivity and electrochemical catalytic activity. Subsequently, upon sequential modification with cytokine Ab1, BSA, cytokine standard solution and AP-Ab2, the resistance in the Nyquist plot gradually increased correspondingly, while the CV peak current gradually decreased, suggesting that the proteins hindered charge transfer at the electrode-solution interface due to increased surface coverage by non-conductive molecules. Furthermore, during DPV measurement, a distinct oxidation peak was observed at approximately 0.15 V after incubation with a target antigen concentration of 10 ng mL^− 1^, indicating the successful formation of a sandwich-type immunocomplex due to antigen-antibody interactions (Fig. 2C).

Moreover, our study underlines the pivotal role of Ti_3_C_2_Tx MXene/Au NPs in the performance of the nanobiosensor. Transmission electron microscopy (TEM) characterization confirmed the layered (Ti_3_C_2_Tx MXene) and spherical (Au NPs) morphologies, as visualized in Fig. 2D–F. The elemental composition of Ti_3_C_2_Tx MXene/Au NPs was further analyzed via energy-dispersive X-ray spectroscopy (EDX) (Fig. 2G), revealing the presence of C, O, Ti, F, Au, and Al. Notably, F and Al originated from residual impurities and were present in significantly lower concentrations compared to C, O, Au, and Ti. The morphology of Ti_3_C_2_Tx MXene was further investigated using atomic force microscopy (AFM). As shown in Fig. 2H, the AFM image displayed flakes with a thickness of approximately 4 nm, corresponding to a three-layer thickness. Compared to GCE and other nanomaterial-modified electrodes, only Ti_3_C_2_Tx MXene presented observable electrocatalytic activity for 1-naphthol oxidation (Fig. 2I), which further demonstrates the unique and ultra-high electrocatalytic activity of Ti_3_C_2_Tx MXene to facilitate the electrode conductivity and the efficiency of charge transfer and transport for the oxidation of 1-naphthol.

### Detection of multi-cytokines with the portable nanobiosensor

**Fig. 3:**
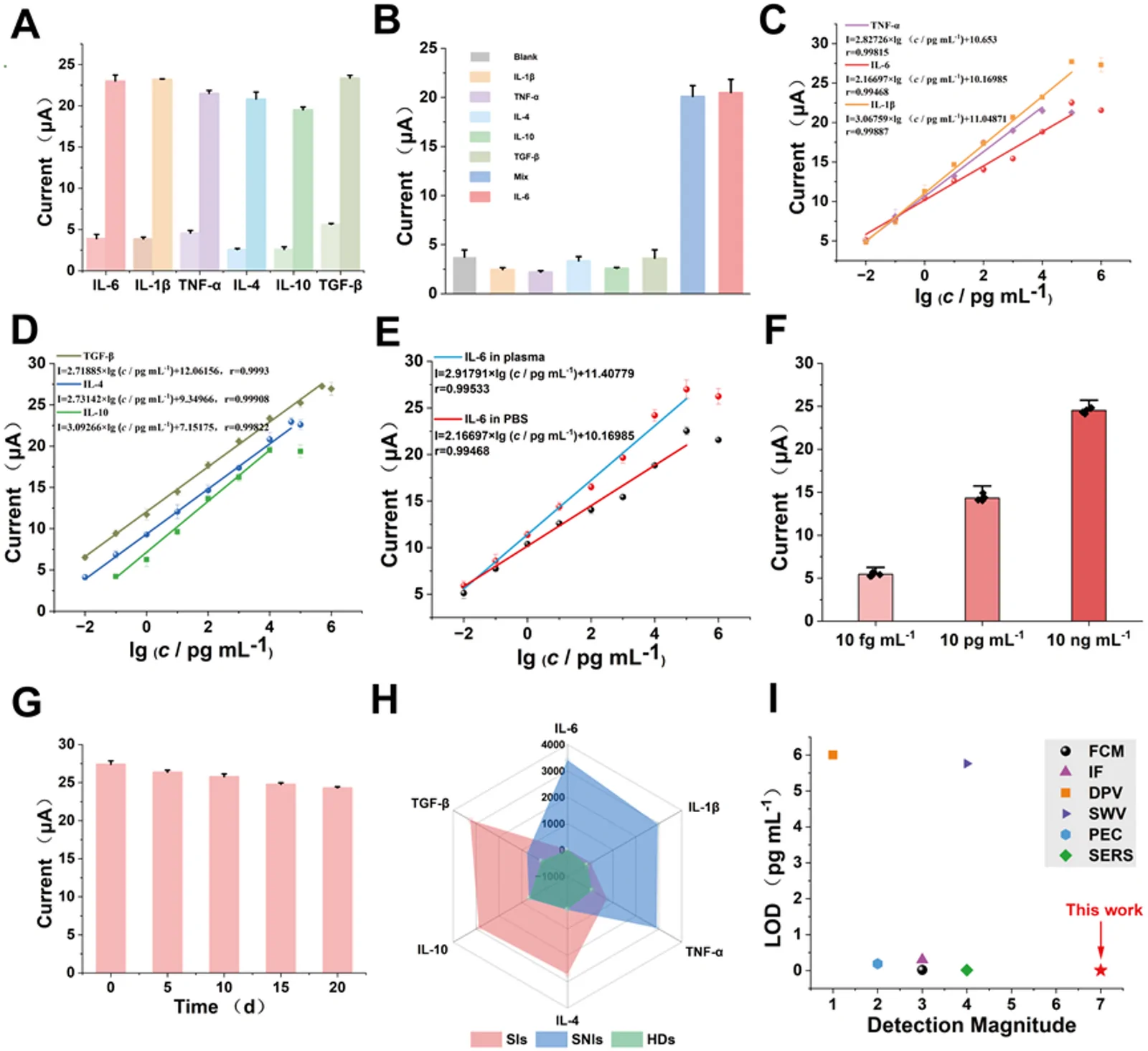
(**A** and **B**) NLISA analysis of reaction substrate deficiency experiments (**A**) and anti-interference experiments (**B**) to confirm the high selectivity of nanobiosensor for the detection of multi-cytokines. (**C**) Evaluation of the sensitivity of the nanobiosensor for inflammatory cytokines detection in PBS. (**D**) Evaluation of the sensitivity of the nanobiosensor for anti-inflammatory cytokines detection in PBS. (**E**) Evaluation of the sensitivities of the nanobiosensor by detecting IL-6 in PBS and plasma respectively. (**F**) Reproducibility of the designed nanobiosensor: Current responses of five independent measurements with 10 fg mL^− 1^, 10 pg mL^− 1^, and 10 ng mL^− 1^ target IL-6. (**G**) Stability of the proposed nanobiosensor. (**H**) The radar plot shows six cytokines from the three different types of samples, including SIs, SNIs, and HDs. (**I**) Comparison between the nanobiosensor and other reported cytokine detection strategies, such as flow cytometry (FCM), immunofluorescence (IF), differential pulse voltammetry (DPV), square wave voltammetry (SWV), photoelectrochemical (PEC), and surface-enhanced Raman spectroscopy ༈SERS༉. Error bars indicate mean ± standard deviation (s.d.)

To validate the capability of the nanobiosensor to accurately detect Multi-Cytokines based on NLISA, we conducted separate experiments on the target substrate deficiency of various cytokines and anti-interference experiments with IL-6 as the target analyte. As shown in Fig. 3A, a distinct current signal can be detected only when all NLISA target substrates are present. Figure 3 C illustrates the anti-interference ability of the nanobiosensor. DPV detection was performed using 1 ng mL^− 1^ IL-6, 100-fold mass ratio (100 ng mL^− 1^) of interfering substances (IL-1β, TNF-α, IL-4, IL-10, TGF-β), and a mixture containing 100 ng mL^− 1^ of interfering substances along with 1 ng mL^− 1^ IL-6. Under identical experimental conditions, the current responses of the interfering substances did not significantly differ from those of the blank solution. Furthermore, the signal response of the mixture remained unchanged compared to that of IL-6 alone. Fig. S3C shows the DPV responses of 1 ng mL^− 1^ IL-6 in PBS, icteric plasma, hemolyzed plasma, lipemic plasma, hyperproteinemic plasma, and hypercellular plasma, respectively. All these results demonstrate that the proposed nanobiosensor possesses excellent selectivity and robust anti-interference ability.

To evaluate the analytical performance of the nanobiosensor, several critical experimental conditions were optimized. The optimized concentrations of anti-IL-6, AP-Ab2, antigen-antibody immobilization time, and DEA buffer pH reached 10 µg mL^− 1^, 5 µg mL^− 1^, 60 min, and 9.6, respectively (Fig. S3A). Under the optimal detection conditions, the sensitivity of the nanobiosensor towards various cytokines was assessed by varying the concentration of cytokine standard solutions in PBS. As shown in Fig. 3C and D, strong linear calibration curves were observed between the corresponding calibration plots of peak currents and the logarithm of target cytokine concentrations. Based on three times the value of the mean standard deviation from blank sample detection, the LODs were determined to be 3.8 fg mL^− 1^ for IL-6, 7.6 fg mL^− 1^ for IL-1β, 15.4 fg mL^− 1^ for TNF-α, 4.6 fg mL^− 1^ for IL-4, 71.4 fg mL^− 1^ for IL-10, and 5.6 fg mL^− 1^ for TGF-β. The optimized experimental conditions for other cytokine-specific assays and analytical performance of the proposed nanobiosensor are provided in Supporting Material. Given the high sensitivity of this proposed nanobiosensor, this approach should be capable of directly detecting cytokines in plasma samples. To validate this hypothesis, we used the nanobiosensor to directly analyze healthy clinical plasma samples spiked with different concentrations of target IL-6 (Fig. 3E). The analysis results after spiking IL-6 into plasma were comparable to those obtained by spiking IL-6 into PBS, with a similar detection range and LOD (9.2 fg mL^− 1^ versus 3.8 fg mL^− 1^). Furthermore, Supporting Table S1 summarizes the detection results of Multi-Cytokines in clinical samples. Satisfactory recovery values for IL-6 were obtained, ranging from 94.0% to 117.8%, with relative standard deviations (RSDs) between 0.46% and 1.29%. Under identical experimental conditions, the same concentration of IL-6 was repeatedly measured using different batches of nanobiosensor, and the results are shown in Fig. 3F. The RSD values were 3.7%, 2.5%, and 1.2%, respectively, indicating acceptable repeatability of the nanobiosensor. Additionally, to investigate the stability of the proposed nanobiosensor, the prepared electrochemical biosensor was stored at 4 °C prior to measurement. After 5 days of storage, there was no significant difference in current response, which was only 3.8% lower than the initial response. After 20 days of storage, the biosensor retained 88.7% of its initial current response (Fig. 3G), demonstrating good stability of the proposed biosensor. The reproducibility and stability performance of the proposed nanobiosensor for detecting other cytokines are detailed in Supporting Material.

In addition, we characterized the performance of the nanobiosensor by measuring the abundance of Multi-Cytokines derived from two different types of samples (SIs and SNIs) and HDs. As shown in Fig. 3H, experimental results exhibited that the expression of Multi-Cytokines in SIs and SNIs was significantly higher than that in HDs, indicating significant heterogeneity. Moreover, anti-inflammatory cytokines were predominantly present in SIs, while inflammatory cytokines were mainly found in SNIs, which is consistent with theoretical knowledge. Figure 3I and Supporting Table S2 compare the nanobiosensor with other reported cytokine detection methods. This proposed method exhibited a lower LOD and a wider detection range, with a larger order of magnitude in detection (Detection Magnitude), highlighting the advantages of NLISA and cascade catalytic strategies, including high sensitivity and accuracy, rapid analysis, and ease of operation.

### Validating nanobiosensor with clinical patient samples

**Fig. 4:**
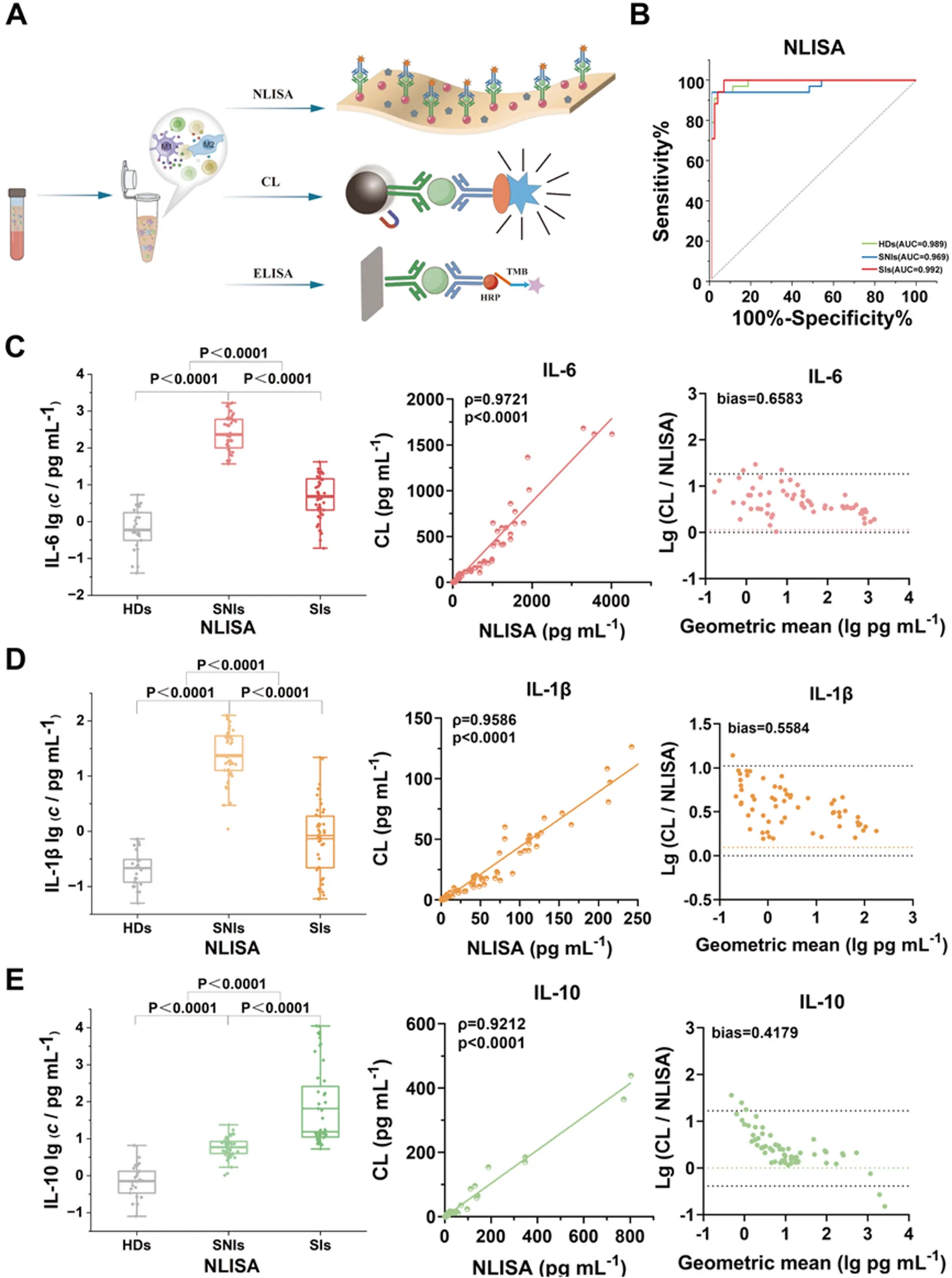
Verification of the ability of the NLISA to enhance the differential diagnosis of SIs, SNIs and HDs. (**A**) Schematic illustration of the multi-cytokines detection in plasma samples using the NLISA, CL, and ELISA assays. (**B**) ROC curves and AUC values of NLISA for multi-cytokines levels in differentiating between SIs, SNIs, and HDs groups. (**C**-**E**) The NLISA-based nanobiosensor demonstrates significant capability in distinguishing cohorts of SIs, SNIs, and HDs in terms of IL-6, IL-1β, and IL-10 levels. Furthermore, a strong correlation between the conventional clinical methods and the NLISA-based nanobiosensor platform was observed, with Spearman’s ρ values greater than 0.85 when analyzing plasma samples. Bland-Altman difference plots (*n* = 60) (IL-6: mean difference: 0.6583, 95% CI: 0.0542 to 1.2620; IL-1β: mean difference: 0.5584, 95% CI: 0.0950 to 1.0220; IL-10: mean difference: 0.4179, 95% CI: −0.3872 to 1.2230)

The clinical translation of POCT based on the NLISA nanobiosensor requires rigorous clinical sample validation. In this study, 60 plasma samples (20 from the SIs, SNIs and HD groups, respectively) were tested using the NLISA nanobiosensor and compared with results from conventional clinical assays (CL and ELISA) (Fig. 4A). We used Spearman rank correlation to evaluate inter-method correlation, and all cytokines yielded Spearman’s ρ > 0.85 (Fig. 4C–E, Fig. S9B–D). Intraclass correlation coefficients (ICCs) were further calculated to assess inter-method agreement, with all ICC values above 0.90, confirming excellent concordance between the two detection assays. Bland-Altman analysis of log-transformed measurements revealed acceptable agreement between the NLISA platform and conventional assays, with a consistent positive systematic bias (conventional readings higher than NLISA) confirmed by nearly all data points lying above the zero-bias line. Notably, the 95% limits of agreement (LoA) fell within the pre-defined acceptable error range, supporting reliable concordance despite this fixed offset. Although NLISA yielded generally lower absolute values, the high correlation and stable bias ensure reliable reflection of cytokine profiles and immune phenotype discrimination.

The box plots in Fig. 4C-E and Fig. S9B-D represent the capability of the nanobiosensor to differentiate SIs, SNIs, and HDs cohorts in terms of IL-6, IL-1β, TNF-α, IL-4, IL-10, and TGF-β levels. Significant differences in cytokine levels among the three cohorts were observed and confirmed by two-tailed Mann-Whitney U statistical analysis with Bonferroni correction. The NLISA-based logistic regression model was constructed using normalized input data to eliminate scale disparities among cytokines, followed by SHAP analysis to interpret the contribution of each cytokine to the classification output (Fig. S9A). As shown in Fig. 4B, the NLISA assay demonstrated diagnostic performance comparable to traditional clinical detection methods, with AUC values of 0.992 for SIs, 0.969 for SNIs, and 0.989 for HDs, respectively.

In summary, the nanobiosensor detection exhibits substantial advantages, offering a simplified workflow and the ability to distinguish all six biomarkers in SIs, SNIs, and HDs patient samples. The distinct cytokine profiles among the cohorts highlight the potential clinical utility of this technology for early identification of sepsis immunoparalysis.

### Clinical differential diagnosis of SIs, SNIs, and HDs using nanobiosensors combined with machine learning

Although nanobiosensors for Multi-Cytokines analysis show promise for early identification of SIs, enhancing the accuracy of differentiating SIs, SNIs, and HDs remains crucial. Single cytokines failed to achieve sufficient sensitivity, specificity, and accuracy (Fig. S10A and Supporting Table S5), and conventional clinical indicators, including C-reactive protein (CRP), procalcitonin (PCT), serum albumin (SA), and total lymphocyte count (TLC), yielded even lower AUCs (Fig. S10B and Supporting Table S6–S7). These results indicate that neither single cytokines nor conventional clinical indicators can reliably distinguish these three groups.

To address this, we utilized machine learning for Multi-Cytokines analysis. We collected 300 clinical samples (100 HDs, 100 SNIs, 100 SIs) and partitioned them into training and validation sets (7:3) via stratified random sampling for reproducibility. Six cytokines (IL-6, IL-1β, TNF-α, IL-4, IL-10, TGF-β) were included as features; no additional clinical indicators were included in the current model. All six cytokines showed significant intergroup differences (overall three-group comparison, *p* < 0.05; Supporting Table S3). Min-max normalization was applied to all features, with parameters fitted exclusively on the training set to prevent data leakage. No missing values were observed. The primary ranking metric was the multiclass macro AUC on the validation set. When AUC values were comparable across models, stability and overfitting risk were further assessed using 5-fold cross-validation, along with cross-validation standard deviation, the training–validation AUC gap, Log Loss, and Brier score. A training–validation AUC gap exceeding 0.15 was flagged as indicative of substantial overfitting risk (Supporting Table S9).

**Fig. 5:**
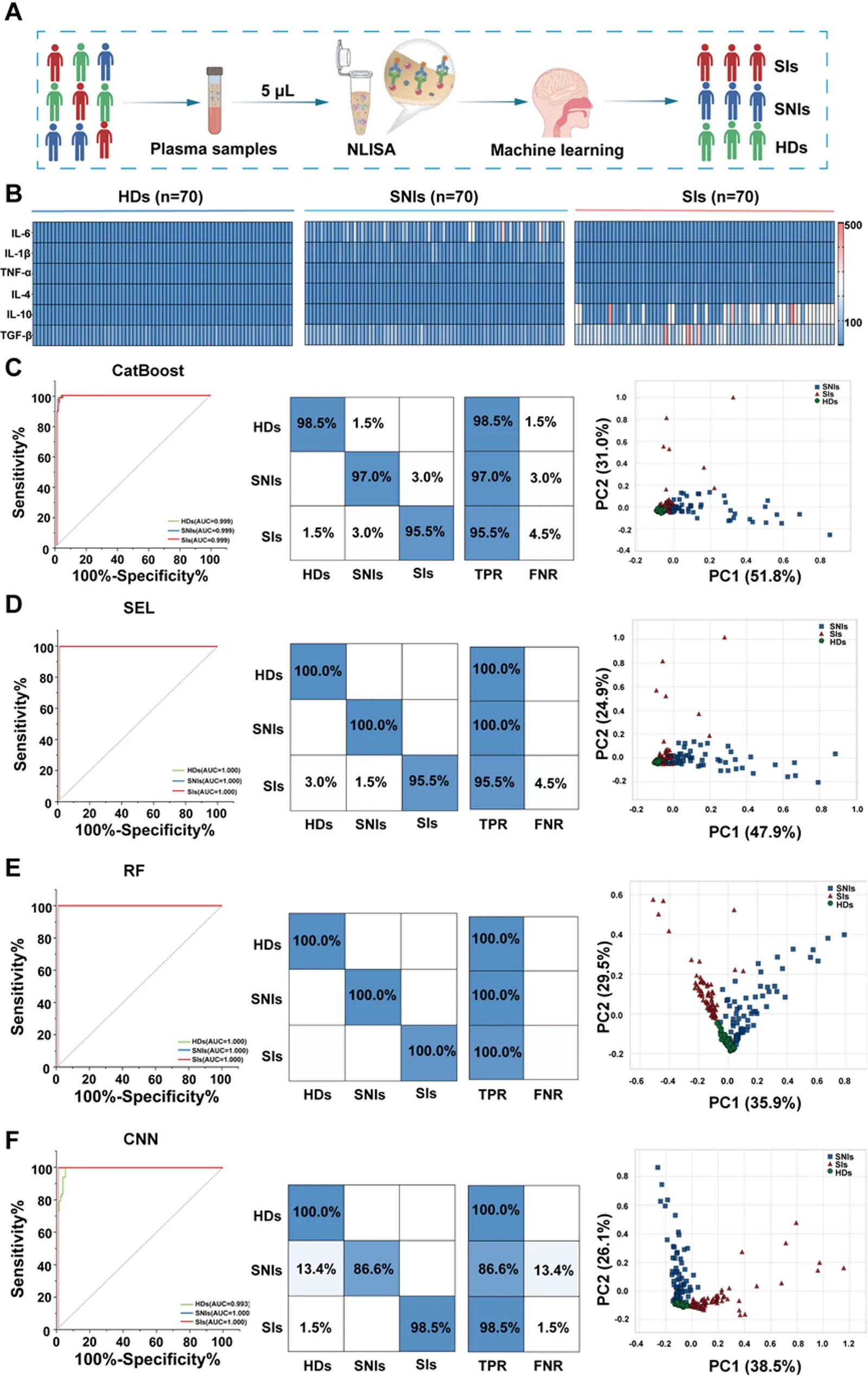
NLISA-based nanobiosensors for multi-cytokines analysis to differentiate SIs, SNIs, and HDs in the training cohort. (**A**) Schematic illustration of nanobiosensors integrated with machine learning for discriminating SIs, SNIs, and HDs. (**B**) Heatmap of multi-cytokines profiles from 70 patients with SI, 70 patients with SNI, and 70 healthy donors. (**C**-**F**) ROC curves, AUC values, diagnostic accuracies, and PCAs for SIs, SNIs, and HDs discrimination based on the CatBoost, SEL, RF, and CNN algorithms

To comprehensively characterize inter-cytokine interactions and provide flexible, personalized diagnostic options, we systematically evaluated multiple machine learning algorithms across cytokine panels of varying dimensionality. Specifically, three-cytokine combinations were modeled using Categorical Boosting (CatBoost), Extreme Gradient Boosting (XGBoost), and Gradient Boosting Decision Tree (GBDT) (Fig. 5C and Fig. S12); four-cytokine combinations were modeled using Stacking Ensemble Learning (SEL), Naive Bayes (NB), and One-dimensional Recurrent Neural Network (RNN) (Fig. 5D and Fig. S13); five-cytokine combinations were modeled using Random Forest (RF), Residual Network for Tabular Data (RNT), and Multilayer Perceptron (MLP) (Fig. 5E and Fig. S14); and the full six-cytokine panel was modeled using One-dimensional Convolutional Neural Network (CNN), Dense Convolutional Network for Tabular Data (DNT), and Gated Recurrent Unit Network (GRU) (Fig. 5F and Fig. S15). This multi-algorithm, multi-panel strategy enables both mechanistic insight into cytokine synergy and adaptable clinical deployment. Detailed descriptions of these algorithms are provided in Supporting Table S4.

The heatmap illustrates the abundance of Multi-Cytokines for each subject in the training cohort (Fig. 5B). Notably, Multi-Cytokines exhibit highly heterogeneous levels in patients with SIs and SNIs. By integrating diverse cytokine panels with corresponding machine learning algorithms, we achieved highly accurate discrimination among SIs, SNIs, and HDs, as evidenced by confusion matrices and further corroborated by ROC curve analyses. Additionally, principal component analysis (PCA) was employed to visualize the underlying data distribution, while SHAP summary plots were used to quantify the relative contribution of each cytokine to the diagnostic predictions (Fig. S11). Collectively, this integrated Multi-Cytokines machine learning framework provides a robust and scalable approach for the precise stratification of SIs, bridging the gap between biomarker discovery and clinical translation.

### Clinical validation cohort

**Fig. 6:**
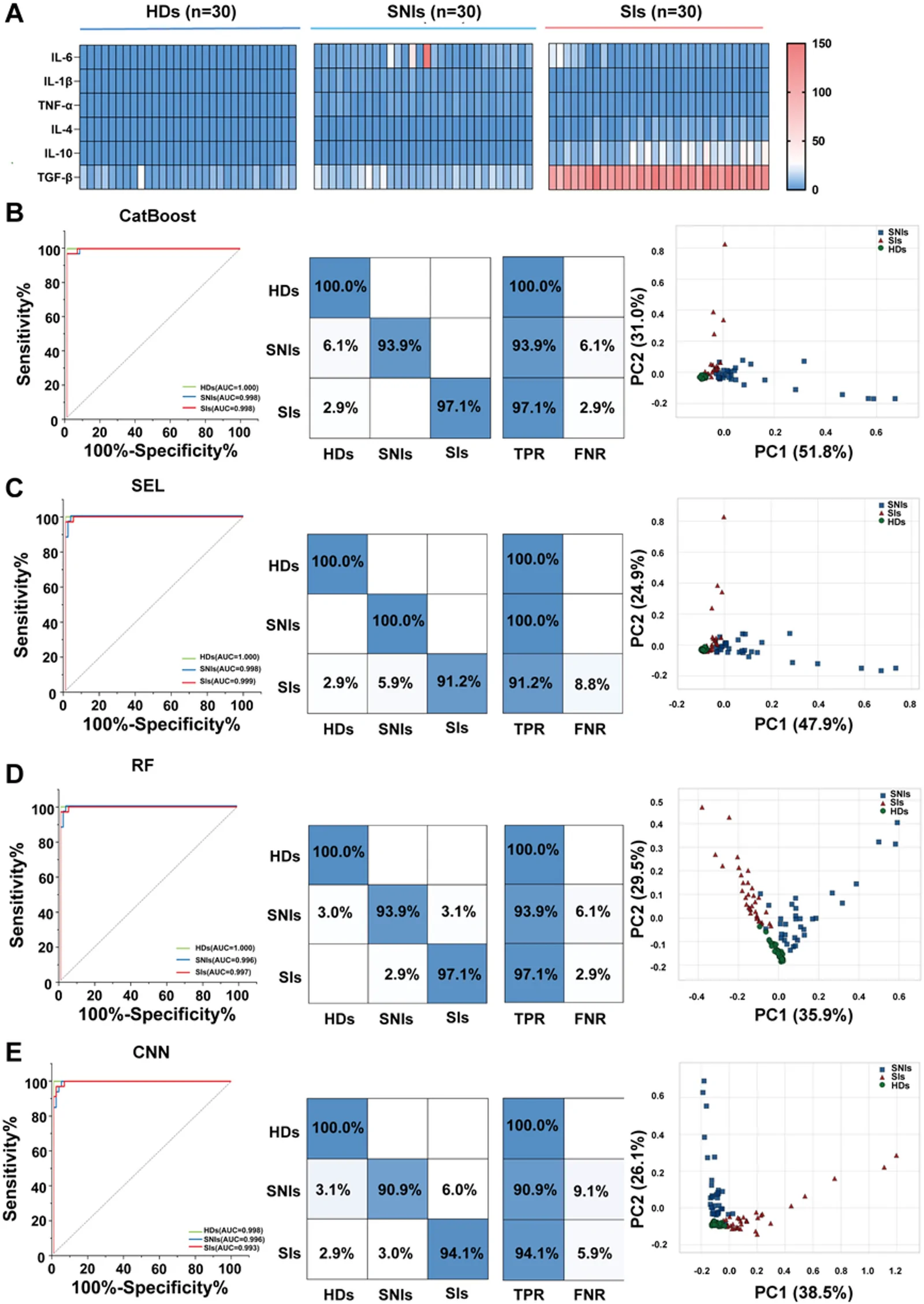
NLISA-based nanobiosensors for multi-cytokines analysis to differentiate SIs, SNIs, and HDs in the validation cohort. (**A**) Heatmap of multi-cytokines profiles from 30 patients with SI, 30 patients with SNI, and 30 healthy donors. (**B**-**E**) ROC curves, AUC values, diagnostic accuracies, and PCAs for SIs, SNIs, and HDs discrimination based on the CatBoost, SEL, RF, and CNN algorithms

Given the promising results obtained in the training cohort, each cytokine combination from the validation cohort was paired with its corresponding model trained on the same cytokine panel from the training cohort for evaluation. The independent validation cohort (*n* = 90, comprising 30 SIs, 30 SNIs, and 30 HDs) was imported into each respective trained model, and all models consistently achieved excellent diagnostic performance in discriminating among the three groups (Fig. 6B-E and Fig. S18-S22). Figure 6A summarizes the abundance of the Multi-Cytokines for each subject, indicating significant heterogeneity across patients. Fig. S16 highlights significant differences in Multi-Cytokines expression among these groups. Statistical significance was assessed using two-tailed Mann-Whitney U test with Bonferroni correction; *p* < 0.05 was considered significant. In addition, the Multi-Cytokines significantly improved the ability to differentiate SIs, SNIs, and HDs compared to single cytokines (Fig. S17). These results demonstrate that regardless of the specific cytokine panel used, the corresponding trained model reliably and accurately distinguished SIs, SNIs, and HDs, underscoring the broad applicability and practicality of the nanobiosensor assay combined with machine learning models for sepsis triage.

## Conclusions

In summary, we developed a portable nanobiosensor based on an NLISA strategy for ultrasensitive and specific analysis of Multi-Cytokines, and integrated machine learning algorithms to construct early identification models for sepsis immunoparalysis. The Ti_3_C_2_Tx MXene/Au NPs nanozyme enhances signal transduction by enlarging the electroactive surface area of the working electrode (WE) and improving charge transfer efficiency. Compared with previously reported analysis technologies, the nanobiosensor offers several advantages: (1) It leverages a specific affinity-based transduction mechanism and the MXene nanozyme cascade amplification effect to construct NLISA, enabling the ultrasensitive detection of IL-6, IL-1β, TNF-α, IL-4, IL-10, and TGF-β. (2) The rapid response afforded by the nanobiosensor facilitates timely clinical decision-making, enabling clinicians to intervene within the critical “golden hour” and initiate appropriate treatment. This prompt action is crucial in mitigating the progression of irreversible organ failure and subsequent mortality associated with delayed responses. (3) Benefiting from its compact structure and simple operation, the nanobiosensor can be flexibly applied in emergency departments and bedside clinical scenarios. Furthermore, the ultra-low sample volume requirement (30 µL) encourages repeated single-point sampling and frequent on-site detection within a single day. Multiple intermittent independent detections further facilitate dynamic evaluation of patients’ real-time immune status and sepsis severity at different time points. (4) Additionally, machine learning models constructed from different cytokine combinations can address diverse clinical needs and cost-effectiveness considerations, enabling personalized diagnostic strategies tailored to distinct patient populations and resource-constrained settings. Such versatility is pivotal in providing a precise immune fingerprint for individual patients, fostering advancements in precision medicine. By importing the validation set data into the respective trained models, all cytokine-model combinations consistently achieved excellent discrimination among SIs, SNIs, and HDs with AUC values greater than 0.95 in the training cohort (*n* = 210) and robust accuracy in the validation cohort (*n* = 90), surpassing single cytokine signals and conventional clinical indicators such as CRP, PCT, total lymphocyte count, and serum albumin.

Nevertheless, several limitations should be acknowledged. First, the recovery rates for Multi-Cytokines in clinical plasma samples ranged from 90.0% to 118.0%, slightly exceeding the conventional acceptance range of 85.0%–115.0%. This deviation, defined as the ratio of measured to spiked concentration, is closely linked to the matrix effect of plasma, as evidenced by the ~35% reduction in calibration slope between plasma and PBS (LOD: 9.2 fg mL⁻¹ in plasma vs. 3.8 fg mL⁻¹ in PBS). Both phenomena arise primarily from nonspecific adsorption and partial signal suppression caused by the complex protein composition of plasma. To address this, matrix-matched calibration or standard addition protocols are recommended for future clinical translation to compensate for plasma-induced signal variations and ensure accurate quantification in real-world samples. Second, although the machine learning models demonstrated excellent diagnostic performance, the sample size remains relatively limited, which may constrain the generalizability of the models. Larger-scale, multicenter studies are warranted to further consolidate the robustness and clinical applicability of these models.

## Supplementary Information

Supplementary Material 1

## Data Availability

No datasets were generated or analysed during the current study.
