# Supplementary material for: A portable nanobiosensor with machine learning: enabling multi-cytokines point-of-care testing and early identification of sepsis immunoparalysis
Source: J Nanobiotechnology. 2026 Aug 14;24:845. doi: 10.1186/s12951-026-04939-5 (PMC13551774; doi:10.1186/s12951-026-04939-5)
Supplement: Supplementary file 1 [file 12951_2026_4939_MOESM1_ESM.docx]

**Supporting Material**

**A portable nanobiosensor with machine learning: Enabling Multi-Cytokines point-of-care testing** **and early identification of sepsis immunoparalysis**

Rongjun Yu^1,3,†^, Jiangling Wu^1, †^, Anyi Chen^2, †^, Jianjiang Xue^1^, Sihan Guo^1^, Siling Chen^1^, Shanshan Dong^1^, Li Li^1^, Xiao Gui^1^, LiLin Wang^1^, Gang Tian^5^, Gang Liu^4,^*, Jingfu Qiu^2,^*, Weixian Chen^3,^*

^1^ *Department of Clinical Laboratory and Medical Sciences Research Center, University-Town Hospital of Chongqing Medical University, Chongqing, 401331, P. R. China*

^2^ *School of Public Health and Management, Chongqing Medical University, Chongqing, 400016, P. R. China*

^3^ *Department of Laboratory Medicine, The Second Affiliated Hospital of Chongqing Medical University, Chongqing 400016, P. R. China*

^4^ *Department of Emergency, Yongchuan Hospital of Chongqing Medical University, Chongqing, 402160, P. R. China*

^5^ *Department of Laboratory Medicine, The Affiliated Hospital of Southwest Medical University, Sichuan Province Engineering Technology Research Center of Molecular Diagnosis of Clinical Diseases, Molecular Diagnosis of Clinical Diseases Key Laboratory of Luzhou, Sichuan, 646000, P. R. China*

* Corresponding authors

E-mail addresses: chenweixian@cqmu.edu.cn, jfqiu@126.com, lg@cqmu.edu.cn.

^†^ These authors contributed equally to this work.

**Experimental section**

**Buffers**

The buffers and solutions involved in this experiment were as follows: DEA buffer (0.1 M diethanolamine, 100 mM KCl and 1 mM MgCl_2_, pH 9.6) and detection buffer (0.1 M DEA buffer containing 7.0 mM 1-naphthyl phosphate, pH 9.6). Washing buffer (Phosphate-buffered saline containing 0.1 M Na_2_HPO_4_, 0.1 M KH_2_PO_4_ and 0.1 M KCl, pH 6.8). The cyclic voltammetry (CV) and electrochemical impedance spectroscopy (EIS) were implemented in 5 mM [Fe (CN)_6_]^3-/4-^ containing 0.1 M KCl. Ultrapure distilled water (18.2 MΩ/cm) obtained from a Millipore Mill-Q purification system was used for all solution preparation.

**Apparatus**

Electrochemical measurements, including differential pulse voltammetry (DPV), CV and EIS were implemented on BIOSYS smart biosensor system (Shenzhen Refresh Intelligent Technology Co., Ltd.). The three-electrode system, which consisted of a saturated calomel electrode (reference electrode，RE), a platinum wire (counter electrode，CE) and a 3-mm diameter glassy carbon electrode (working electrode，WE), was used. Chemiluminescence assays (CL) were measured by C2000 Fully Automatic Immune Analyzer (Beijing Hotgen Biotech Co., Ltd). Flow cytometry analysis was performed using a CytoFLEX LX flow cytometer (Beckman Coulter, USA). Transmission electron microscopy (TEM) and Atomic force microscopy (AFM) images were recorded by a Bruker Dimension Icon microscope (Germany). Energy dispersive X-ray spectroscopy (EDX) data was obtained using a JEOL JSM-6700F microscope (Japan).

**Synthesis of Au NPs**

In short, sodium citrate (2 mL, 1%) was rapidly added into the boiling solution of HAuCl_4_ (100 mL, 0.01%) under magnetic stirring and maintained at 100 °C for 15 min. After the color changed from gray to wine-red, the mixture was then cooled to room temperature with continued stirring. In this way, the Au NPs had an average particle size of 15 nm.

**Electrochemical measurement**

The CV measurements were carried out by scanning from -0.2 V to 0.6 V at a scan rate of 100 mV s^−1^. The EIS parameters contained a 10 mV amplitude and a frequency sweep range from 10^−1^ Hz to 10^5^ Hz. The DPV measurements were executed with voltages within the range of -0.05 ~ 0.55 V, modulation amplitudes of 0.07 V, modulation time of 0.05 s and interval time of 0.2 s. All measurements were obtained under ambient conditions at room temperature (25 ± 1 °C).

**Specific inclusion and exclusion criteria of patient selection**

The SIs classification system established in this study adopts routine ICU indicators with favorable clinical practicality and reproducibility. These biomarkers are rapidly and readily detectable, which facilitates the early screening and identification of SIs.

Inclusion criteria for sepsis patients: Diagnosis must be based on the new Sepsis-3.0 definition, which entails an increase in the Sequential Organ Failure Assessment (SOFA) score due to infection and a quick SOFA (qSOFA) score of ≥2 points. The qSOFA criteria include: (1) Respiratory rate ≥22 breaths/min; (2) Altered mental status; (3) Systolic blood pressure ≤100 mmHg. Each criterion contributes 1 point to the qSOFA score.

Based on confirmed sepsis diagnostic criteria, the inclusion and exclusion criteria for patients with sepsis immunoparalysis were established according to authoritative international sepsis guidelines, published literature [1-5], and cumulative clinical experience. Patients meeting any one of the criteria below, in combination with clinical treatment status assessment, are eligible: (1) C-reactive protein (CRP) >150 mg L^-1^ with a persistently declining trend; (2) Procalcitonin (PCT) >0.50 ng mL^-1^ with a persistently declining trend [6-8]; (3) Immunosuppression: Total lymphocyte count (TLC) <0.80×10^9^ L^-1^ with a persistently declining trend [9-11]; (4) Catabolism, defined by any one of the following: Serum albumin (SA) <30 g L^-1^; Creatinine height index <80%; Weight loss >10% during hospitalization or BMI <18 kg m^2^ [12-15].

Rather than relying on a single indicator for classification, our comprehensive diagnostic strategy integrates multiple laboratory parameters, clinical manifestations, and immunotherapy status of patients to ensure optimal classification accuracy. Furthermore, we performed flow cytometric detection of monocytic human leukocyte antigen-DR (HLA-DR) expression in a subset of samples from the SIs, SNIs and HDs groups [16, 17]. The results showed that monocytic HLA-DR expression was significantly downregulated in the SIs group, with the proportion of HLA-DR-positive monocytes below 30% (Fig. S1). This finding is fully consistent with the classic diagnostic criteria for SIs specified in current authoritative guidelines, further verifying the reliability of our classification strategy.

Exclusion criteria: (1) Patients with malignant tumors; (2) Individuals with hematological disorders; (3) Patients with severe liver disease and significant blood loss; (4) Those who received platelet transfusions within 48 hours prior to diagnosis; (5) Individuals who received heparin preparations within one week prior to diagnosis; (6) Patients who underwent blood purification therapy within one week prior to diagnosis; (7) Those who recently received immunosuppressive therapy.

Although our comprehensive classification criteria are supported by published evidence and HLA-DR validation results, we acknowledge that the sample size for flow cytometry analysis is limited and cannot fully represent the immunological characteristics of the entire SIs cohort. Classification based on routine clinical indicators, instead of comprehensive immune monitoring, may inevitably lead to misclassification and subsequent alterations in diagnostic sensitivity and specificity. In the future, we will incorporate the latest guidelines to conduct large-sample, multicenter cohort studies and adopt standardized immune monitoring techniques, including monocytic HLA-DR detection and ex vivo cytokine functional assays, to further validate the accuracy and stability of our findings in the overall study population.

**Fig.S1** Flow cytometric detection of HLA-DR expression on CD14⁺ monocytes in peripheral blood of HDs, SIs and SNIs; (C) HLA-DR expression was significantly different between HDs, SNIs and SIs.

**Optimization of experimental conditions and analytical performance of the proposed nanobiosensor**

To estimate analytical performance of the nanobiosensor, the current responses toward IL-6 at different concentrations were recorded under the optimal conditions through DPV measurements. As shown in Fig. S3B, the detection signal increased with the increasing concentration of target IL-6.

The optimized experimental conditions for other cytokine-specific assays are as follows: the optimized concentrations of anti-IL-1β, AP-Ab2, antigen-antibody immobilization time, and DEA buffer pH reached 5 μg mL^-1^, 5 μg mL^-1^, 45 min, and 9.6, respectively (Fig. S4A). The optimized concentrations of anti-TNF-α, AP-Ab2, antigen-antibody immobilization time, and DEA buffer pH reached 15 μg mL^-1^, 10 μg mL^-1^, 60 min, and 9.6, respectively (Fig. S5A). The optimized concentrations of anti-IL-4, AP-Ab2, antigen-antibody immobilization time, and DEA buffer pH reached 10 μg mL^-1^, 10 μg mL^-1^, 45 min, and 9.6, respectively (Fig. S6A). The optimized concentrations of anti-IL-10, AP-Ab2, antigen-antibody immobilization time, and DEA buffer pH reached 10 μg mL^-1^, 5 μg mL^-1^, 60 min, and 9.6, respectively (Fig. S7A). The optimized concentrations of anti-TGF-β, AP-Ab2, antigen-antibody immobilization time, and DEA buffer pH reached 5 μg mL^-1^, 5 μg mL^-1^, 45 min, and 9.6, respectively (Fig. S8A).

The reproducibility and stability performance of the proposed nanobiosensor for detecting other cytokines are as follows: under identical experimental conditions, the same concentration of cytokines was repeatedly measured using different batches of nanobiosensors. The relative standard deviation (RSD) values of IL-1β were 2.7%, 1.4%, and 0.8% (Fig. S4D). The RSD values of TNF-α were 3.9%, 1.5%, and 1.5% (Fig. S5D). The RSD values of IL-4 were 3.8%, 3.1%, and 0.6% (Fig. S6D). The RSD values of IL-10 were 4.3%, 1.8%, and 1.4% (Fig. S7D). The RSD values of TGF-β were 3.4%, 2.5%, and 1.1% (Fig. S8D), indicating acceptable repeatability of the nanobiosensor. Additionally, the prepared nanobiosensor was stored at 4 °C prior to measurement. After 5 days of storage, the IL-1β signal response was only 1.5% lower than the initial response. After 20 days of storage, the nanobiosensor retained 88.6% of its initial IL-1β current response (Fig. S4E). After 5 days of storage, the TNF-α signal response was only 1.2% lower than the initial response. After 20 days of storage, the nanobiosensor retained 91.9% of its initial TNF-α current response (Fig. S5E). After 5 days of storage, the IL-4 signal response was only 1.5% lower than the initial response. After 20 days of storage, the nanobiosensor retained 88.9% of its initial IL-4 current response (Fig. S6E). After 5 days of storage, the IL-10 signal response was only 2.8% lower than the initial response. After 20 days of storage, the nanobiosensor retained 90.7% of its initial IL-10 current response (Fig. S7E). After 5 days of storage, the TGF-β signal response was only 1.1% lower than the initial response. After 20 days of storage, the nanobiosensor retained 91.7% of its initial TGF-β current response (Fig. S8E), demonstrating good stability of the proposed nanobiosensor.

**Fig. S2.** (A-D) Significant differences in the expression of IL-1β, TNF-α，IL-4, and TGF-β cytokines among SIs, SNIs, and HDs groups.

**Fig. S3.** (A) Optimization of IL-6 detection experimental conditions. (B) DPV curves response of the nanobiosensor upon the increase in target IL-6 concentration (from a to i: 0, 10 fg mL^-1^, 100 fg mL^-1^, 1 pg mL^-1^, 10 pg mL^-1^, 100 pg mL^-1^, 1 ng mL^-1^, 10 ng mL^-1^, and 100 ng mL^-1^, separately). (C) Anti-interference experiments: DPV responses of 1 ng mL^-1^ IL-6 in PBS (a), icteric plasma (b), hemolyzed plasma (c), lipemic plasma (d), hyperproteinemic plasma (e), and hypercellular plasma (f), respectively. The error bars represent the standard deviation of three parallel measurements.

**Fig. S4.** (A) Optimization of IL-1β detection experimental conditions. (B) DPV curves response of the nanobiosensor upon the increase in target IL-1β concentration (from a to i: 0, 10 fg mL^-1^, 100 fg mL^-1^, 1 pg mL^-1^, 10 pg mL^-1^, 100 pg mL^-1^, 1 ng mL^-1^, 10 ng mL^-1^, and 100 ng mL^-1^, separately). (C) Specificity of the proposed nanobiosensor for 1 ng mL^-1^ of IL-1β against the zero analyte (Blank); other interference cytokines with 100 ng mL^-1^: IL-4, TNF-α, IL-6, IL-10, TGF-β and their mixture (Mix). (D) Anti-interference experiments: DPV responses of 1 ng mL^-1^ IL-1β in PBS (a), icteric plasma (b), hemolyzed plasma (c), lipemic plasma (d), hyperproteinemic plasma (e), and hypercellular plasma (f), respectively. (E) Reproducibility of the designed nanobiosensor: current responses of five independent measurements with 10 fg mL^-1^, 10 pg mL^-1^, and 10 ng mL^-1^ target IL-1β. (F) Stability of the proposed nanobiosensor. The error bars represent the standard deviation of three parallel measurements.

**Fig. S5.** (A) Optimization of TNF-α detection experimental conditions. (B) DPV curves response of the nanobiosensor upon the increase in target TNF-α concentration (from a to h: 0, 10 fg mL^-1^, 100 fg mL^-1^, 1 pg mL^-1^, 10 pg mL^-1^, 100 pg mL^-1^, 1 ng mL^-1^, and 10 ng mL^-1^, separately). (C) Specificity of the proposed nanobiosensor for 1 ng mL^-1^ of TNF-α against the zero analyte (Blank); other interference cytokines with 100 ng mL^-1^: IL-4, IL-1β, IL-6, IL-10, TGF-β and their mixture (Mix). (D) Anti-interference experiments: DPV responses of 1 ng mL^-1^ TNF-α in PBS (a), icteric plasma (b), hemolyzed plasma (c), lipemic plasma (d), hyperproteinemic plasma (e), and hypercellular plasma (f), respectively. (E) Reproducibility of the designed nanobiosensor: current responses of five independent measurements with 10 fg mL^-1^, 10 pg mL^-1^, and 10 ng mL^-1^ target TNF-α. (F) Stability of the proposed nanobiosensor. The error bars represent the standard deviation of three parallel measurements.

**Fig. S6.** (A) Optimization of IL-4 detection experimental conditions. (B) DPV curves response of the nanobiosensor upon the increase in target IL-4 concentration (from a to i: 0, 10 fg mL^-1^, 100 fg mL^-1^, 1 pg mL^-1^, 10 pg mL^-1^, 100 pg mL^-1^, 1 ng mL^-1^, 10 ng mL^-1^, and 50 ng mL^-1^, separately). (C) Specificity of the proposed nanobiosensor for 1 ng mL^-1^ of IL-4 against the zero analyte (Blank); other interference cytokines with 100 ng mL^-1^**)**: IL-1β, TNF-α, IL-6, IL-10, TGF-β and their mixture (Mix). (D) Anti-interference experiments: DPV responses of 1 ng mL^-1^ IL-4 in PBS (a), icteric plasma (b), hemolyzed plasma (c), lipemic plasma (d), hyperproteinemic plasma (e), and hypercellular plasma (f), respectively. (E) Reproducibility of the designed nanobiosensor: current responses of five independent measurements with 10 fg mL^-1^, 10 pg mL^-1^, and 10 ng mL^-1^ target IL-4. (F) Stability of the proposed nanobiosensor. The error bars represent the standard deviation of three parallel measurements.

**Fig. S7.** (A) Optimization of IL-10 detection experimental conditions. (B) DPV curves response of the nanobiosensor upon the increase in target IL-10 concentration (from a to g: 0, 100 fg mL^-1^, 1 pg mL^-1^, 10 pg mL^-1^, 100 pg mL^-1^, 1 ng mL^-1^, and 10 ng mL^-1^, separately). (C) Specificity of the proposed nanobiosensor for 1 ng mL^-1^ of IL-10 against the zero analyte (Blank); other interference cytokines with 100 ng mL^-1^: IL-4, IL-1β, IL-6, TNF-α, TGF-β and their mixture (Mix). (D) Anti-interference experiments: DPV responses of 1 ng mL^-1^ IL-10 in PBS (a), icteric plasma (b), hemolyzed plasma (c), lipemic plasma (d), hyperproteinemic plasma (e), and hypercellular plasma (f), respectively. (E) Reproducibility of the designed nanobiosensor: current responses of five independent measurements with 1 pg mL^-1^, 100 pg mL^-1^, and 10 ng mL^-1^ target IL-10. (F) Stability of the proposed nanobiosensor. The error bars represent the standard deviation of three parallel measurements.

**Fig. S8.** (A) Optimization of TGF-β detection experimental conditions. (B) DPV curves response of the nanobiosensor upon the increase in target TGF-β concentration (from a to j: 0, 10 fg mL^-1^, 100 fg mL^-1^, 1 pg mL^-1^, 10 pg mL^-1^, 100 pg mL^-1^, 1 ng mL^-1^, 10 ng mL^-1^, 100 ng mL^-1^, and 500 ng mL^-1^, separately). (C) Specificity of the proposed nanobiosensor for 1 ng mL^-1^ of TGF-β against the zero analyte (Blank); other interference cytokines with 100 ng mL^-1^: IL-4, IL-1β, IL-6, TNF-α, IL-10 and their mixture (Mix). (D) Anti-interference experiments: DPV responses of 1 ng mL^-1^ TGF-β in PBS (a), icteric plasma (b), hemolyzed plasma (c), lipemic plasma (d), hyperproteinemic plasma (e), and hypercellular plasma (f), respectively. (E) Reproducibility of the designed nanobiosensor: current responses of five independent measurements with 10 fg mL^-1^, 10 pg mL^-1^, and 10 ng mL^-1^ target TGF-β. (F) Stability of the proposed nanobiosensor. The error bars represent the standard deviation of three parallel measurements.

 **Fig. S9.** (A) SHAP feature importance bar plot showing the mean absolute SHAP value of each cytokine. (B-D) The NLISA-based nanobiosensor demonstrates significant capability in distinguishing cohorts of SIs, SNIs, and HDs in terms of TNF-α, IL-4, and TGF-β levels. Furthermore, a strong correlation between the conventional clinical methods and the NLISA-based nanobiosensor platform was observed, with Spearman’s ρ values greater than 0.85 when analyzing plasma samples. Bland-Altman difference plots (n=60) (TNF-α: mean difference: 0.7799, 95% CI: 0.0237 to 1.5360; IL-4: mean difference: 1.318, 95% CI: 0.4415 to 2.195; TGF-β: mean difference: 0.2801, 95% CI: -0.0652 to 0.6254).

**Fig. S10.** (A) ROC curves and AUC values of the IL-6, IL-1β, TNF-α, IL-4, IL-10 and TGF-β for discriminating SIs, SNIs, and HDs. (B) ROC curves and AUC values of CRP, PCT, SA and TLC for SIs, SNIs, and HDs diagnosis.

**Fig. S11.** (A-D) SHAP feature importance bar plots showing the mean absolute SHAP value of each cytokine in CatBoost, SEL, RF, and CNN algorithms.

**Fig. S12.** (A-B) SHAP feature importance bar plots, ROC curves, AUC values, diagnostic accuracies, and PCAs for SIs, SNIs, and HDs discrimination based on XGBoost and GBDT algorithms.

 **Fig. S13.** (A-B) SHAP feature importance bar plots, ROC curves, AUC values, diagnostic accuracies, and PCAs for SIs, SNIs, and HDs discrimination based on NB and RNN algorithms.

 **Fig. S14.** (A-B) SHAP feature importance bar plots, ROC curves, AUC values, diagnostic accuracies, and PCAs for SIs, SNIs, and HDs discrimination based on RNT and MLP algorithms.

 **Fig. S15.** (A-B) SHAP feature importance bar plots, ROC curves, AUC values, diagnostic accuracies, and PCAs for SIs, SNIs, and HDs discrimination based on DNT and GRU algorithms.

 **Fig. S16.** (A-F) Significant differences in the expression of IL-6, IL-1β, TNF-α，IL-4, IL-10 and TGF-β cytokines among SIs, SNIs, and HDs groups in the clinical validation cohort.

**Fig. S17.** (A-F) ROC curves and AUC values of IL-6, IL-1β, TNF-α, IL-4, IL-10, and TGF-β for discriminating SIs, SNIs, and HDs in the clinical validation cohort.

 **Fig. S18.** (A-D) SHAP feature importance bar plots showing the mean absolute SHAP value of each cytokine in CatBoost, SEL, RF, and CNN algorithms in the clinical validation cohort.

 **Fig. S19.** (A-B) SHAP feature importance bar plots, ROC curves, AUC values, diagnostic accuracies, and PCAs for SIs, SNIs, and HDs discrimination based on XGBoost and GBDT algorithms in the clinical validation cohort.

 **Fig. S20.** (A-B) SHAP feature importance bar plots, ROC curves, AUC values, diagnostic accuracies, and PCAs for SIs, SNIs, and HDs discrimination based on NB and RNN algorithms in the clinical validation cohort.

 **Fig. S21.** (A-B) SHAP feature importance bar plots, ROC curves, AUC values, diagnostic accuracies, and PCAs for SIs, SNIs, and HDs discrimination based on RNT and MLP algorithms in the clinical validation cohort.

 **Fig. S22.** (A-B) SHAP feature importance bar plots, ROC curves, AUC values, diagnostic accuracies, and PCAs for SIs, SNIs, and HDs discrimination based on DNT and GRU algorithms in the clinical validation cohort.

**Table S1.** Determination of Multi-Cytokines in clinical samples (n=3) with the developed nanobiosensor.

The detection results for Multi-Cytokines in clinical samples are described as the following: for IL-1β, recovery values ranged from 93.5% to 114.6% with RSDs between 0.21% and 2.79%; for TNF-α, recovery values were between 90.0% and 118.4% with RSDs ranging from 0.22% to 1.91%; for IL-4, recovery values spanned from 90.7% to 113.6% with RSDs between 0.46% and 3.22%; for IL-10, recovery values were from 91.2% to 118.3% with RSDs between 0.31% and 3.37%; and for TGF-β, recovery values ranged from 93.5% to 115.0% with RSDs between 0.14% and 3.28%.

| Samples |  | Added  [pg mL^-1^] | Found  [pg mL^-1^] | Recovery [%] | RSD  [%] |
| --- | --- | --- | --- | --- | --- |
| IL-6 | 1 | 0.05 | 0.047 | 94.0 | 1.29 |
|  | 2 | 0.5 | 0.485 | 97.0 | 1.06 |
|  | 3 | 5 | 5.890 | 117.8 | 1.07 |
|  | 4 | 50 | 48.706 | 97.4 | 0.46 |
|  | 5 | 500 | 509.100 | 101.8 | 0.89 |
|  | 6 | 5000 | 5781.109 | 115.6 | 1.16 |
| IL-1β | 1 | 0.05 | 0.056 | 112.0 | 2.79 |
|  | 2 | 0.5 | 0.504 | 100.8 | 1.28 |
|  | 3 | 5 | 4.675 | 93.5 | 0.43 |
|  | 4 | 50 | 54.617 | 109.2 | 1.35 |
|  | 5 | 500 | 573.158 | 114.6 | 0.21 |
|  | 6 | 5000 | 5250.248 | 105.0 | 0.76 |
| TNF-α | 1 | 0.05 | 0.045 | 90.0 | 1.46 |
|  | 2 | 0.5 | 0.524 | 104.8 | 0.22 |
|  | 3 | 5 | 5.922 | 118.4 | 1.65 |
|  | 4 | 50 | 51.386 | 102.8 | 1.91 |
|  | 5 | 500 | 513.206 | 102.6 | 1.51 |
|  | 6 | 5000 | 5640.729 | 112.8 | 0.99 |
| IL-4 | 1 | 0.05 | 0.052 | 104.0 | 3.22 |
|  | 2 | 0.5 | 0.478 | 95.6 | 0.46 |
|  | 3 | 5 | 5.363 | 107.3 | 0.77 |
|  | 4 | 50 | 45.332 | 90.7 | 0.63 |
|  | 5 | 500 | 568.028 | 113.6 | 1.66 |
|  | 6 | 5000 | 5245.676 | 104.9 | 0.46 |
| IL-10 | 1 | 0.05 | 0.051 | 102.0 | 3.37 |
|  | 2 | 0.5 | 0.468 | 93.6 | 2.73 |
|  | 3 | 5 | 4.560 | 91.2 | 0.54 |
|  | 4 | 50 | 56.071 | 112.1 | 1.63 |
|  | 5 | 500 | 543.180 | 108.6 | 0.94 |
|  | 6 | 5000 | 5914.456 | 118.3 | 0.31 |
| TGF-β | 1 | 0.05 | 0.049 | 98.0 | 2.17 |
|  | 2 | 0.5 | 0.575 | 115.0 | 1.51 |
|  | 3 | 5 | 4.677 | 93.5 | 0.14 |
|  | 4 | 50 | 49.629 | 99.3 | 1.04 |
|  | 5 | 500 | 516.941 | 103.4 | 2.53 |
|  | 6 | 5000 | 4943.141 | 98.9 | 3.28 |

**Table S2.** Comparison of available methods for the detection of cytokine.

| **Methods** | **Sample volume** | **Linear Range [pg** **mL^-1^]** | **LOD [pg mL^-1^]** | **Cytokine**  **Ref.** |
| --- | --- | --- | --- | --- |
| FCM | 20 μL | 6.5 ~ 2×10^3^ | 0.016 | IL-6 [18] |
| IF | 250 pL | 0.3 ~ 312.5  1 ~2500 | 0.3  1 | IL-6  TNF-α [19] |
| DPV | / | 50~ 750 | 14.4 | IL-6 [20] |
| SWV | 5 μL | 5.761~ 2×10^4^ | 5.761 | IL-6 [21] |
| PEC | / | 1~ 100 | 0.19 | IL-6 [22] |
| SERS | / | 0.1~ 1×10^3^ | 0.0124 | IL-6 [23] |
| **DPV** | **5** μL |  |  | **This work** |
|  |  | 0.01 ~ 1×10^5^ | 0.0038 | IL-6 |
|  |  | 0.01 ~ 1×10^5^ | 0.0076 | IL-1β |
|  |  | 0.01 ~ 1×10^4^ | 0.0154 | TNF-α |
|  |  | 0.01 ~ 5×10^4^ | 0.0046 | IL-4 |
|  |  | 0.10 ~ 1×10^4^ | 0.0714 | IL-10 |
|  |  | 0.01 ~ 5×10^5^ | 0.0056 | TGF-β |

FCM: flow cytometry; IF: immunofluorescence; DPV: differential pulse voltammetry; SWV: square wave voltammetry; PEC: photoelectrochemistry; SERS: surface-enhanced raman scattering; LOD: limit of detection.

**Table S3.** Intergroup differences of Multi-Cytokines across HD, SNIs and SIs groups.

| **Feature** | **Comparison** | **P text** | **FDR P text** |
| --- | --- | --- | --- |
| **IL-6** | SNIs vs HDs | <0.001 | <0.001 |
|  | SIs vs HDs | <0.001 | <0.001 |
|  | SIs vs SNIs | <0.001 | <0.001 |
| **IL-1β** | SNIs vs HDs | <0.001 | <0.001 |
|  | SIs vs HDs | <0.001 | <0.001 |
|  | SIs vs SNIs | <0.001 | <0.001 |
| **TNF-α** | SNIs vs HDs | <0.001 | <0.001 |
|  | SIs vs HDs | <0.001 | <0.001 |
|  | SIs vs SNIs | <0.001 | <0.001 |
| **IL-4** | SNIs vs HDs | 0.432 | 0.432 |
|  | SIs vs HDs | <0.001 | <0.001 |
|  | SIs vs SNIs | <0.001 | <0.001 |
| **IL-10** | SNIs vs HDs | <0.001 | <0.001 |
|  | SIs vs HDs | <0.001 | <0.001 |
|  | SIs vs SNIs | <0.001 | <0.001 |
| **TGF-β** | SNIs vs HDs | <0.001 | <0.001 |
|  | SIs vs HDs | <0.001 | <0.001 |
|  | SIs vs SNIs | <0.001 | <0.001 |

**Table S4.** Analysis of machine learning algorithms. Each algorithm has distinctive features and differences, which contribute to its respective classification capability.

| Machine learning algorithms | Functions |
| --- | --- |
| CatBoost | A gradient boosting decision tree algorithm that natively handles categorical features via ordered boosting and symmetric trees. It is robust for small-sample data and effectively captures feature interactions, making it well-suited for biomedical datasets with limited samples and complex dependencies. |
| XGBoost | A regularized gradient boosting decision tree algorithm that incorporates L1 and L2 regularization to control model complexity and prevent overfitting. It is widely used for structured tabular data due to its computational efficiency and strong predictive performance. |
| GBDT | An ensemble learning algorithm that sequentially builds weak decision trees, with each tree correcting the residuals of the previous one. This iterative boosting process progressively improves the model's ability to capture complex decision boundaries. |
| SEL | An ensemble method that combines multiple base models (e.g., logistic regression, random forest, naive Bayes) by feeding their outputs into a meta-learner for final prediction. This two-level architecture leverages the complementary strengths of diverse base models to improve overall predictive accuracy and generalization. |
| NB | A probabilistic classifier based on Bayes' theorem with a strong independence assumption among features. Despite its simplifying assumption, it serves as a fast and effective baseline model, particularly for high-dimensional tabular data. |
| RNN | A recurrent neural network architecture that processes sequential input data in one dimension. It captures temporal dependencies within short feature sequences by maintaining a hidden state that propagates information across time steps, making it suitable for modeling ordered cytokine panels as sequential inputs. |
| RF | An ensemble learning method that builds multiple decision trees trained on random subsets of data and features, with final predictions determined by majority voting. This bagging-based approach effectively reduces the overfitting risk inherent in individual decision trees. |
| RNT | A deep neural network architecture that incorporates residual (skip) connections to mitigate training degradation in deep models. By allowing gradients to bypass layers directly, it enables effective training of deeper networks on structured tabular data. |
| MLP | A basic feedforward neural network composed of multiple fully connected layers with nonlinear activation functions. It serves as a fundamental deep learning model for capturing nonlinear patterns in tabular data for classification tasks. |
| CNN | A convolutional neural network that processes one-dimensional sequential input using convolutional filters along the temporal axis. Unlike image-based 2D-CNN, it extracts local patterns from feature sequences such as ordered biomarker panels. |
| DNT | A deep neural network architecture featuring dense (skip) connections where each layer receives feature maps from all preceding layers. This connectivity pattern promotes feature reuse and alleviates the vanishing gradient problem, enabling efficient training of deeper networks on structured tabular data. |
| GRU | A lightweight recurrent neural network architecture that uses gating mechanisms (reset gate and update gate) to control information flow, similar to LSTM but with fewer parameters and simpler structure. This makes it computationally more efficient while still effectively capturing sequential dependencies. |

**Table S5.** AUCs, sensitivities and specificities of single cytokine for SIs and SNIs diagnosis in the training cohort (n = 210).

|  | **Single cytokine** | **AUC**  **[95%CI]** | **Cutoff** | **Sensitivity [%]**  **[95%CI]** | **Specificity [%] [95%CI]** |
| --- | --- | --- | --- | --- | --- |
| **SIs**  **vs**  **SNIs** | IL-6 | 0.75  （0.66-0.84） | ＜11.95 | 65.71  （54.04-75.75） | 72.86  （61.46-81.88） |
|  | IL-1β | 0.90  （0.85-0.95） | ＜1.38 | 60.00  （48.29-70.67） | 91.43  （82.53-96.01） |
|  | TNF-α | 0.74  （0.66-0.82） | ＜1.01 | 50.00  （38.60-61.40） | 88.57  （79.04-94.09） |
|  | IL-4 | 0.77  （0.69-0.85） | ＞0.66 | 54.29  （42.70-65.43） | 100.00  （94.80-100.00） |
|  | IL-10 | 0.95  （0.92-0.98） | ＞8.95 | 91.43  （82.53-96.01） | 85.71  （75.66-92.05） |
|  | TGF-β | 0.97  （0.95-0.99） | ＞98.33 | 95.71  （88.14-98.83） | 87.14  （77.34-93.09） |
| **SIs**  **vs**  **HDs** | IL-6 | 0.96  （0.93-0.98） | ＞0.81 | 91.43  （82.53-96.01） | 81.43  （70.77-88.81） |
|  | IL-1β | 0.83  （0.76-0.90） | ＞0.17 | 85.71  （75.66-92.05） | 50.00  （38.60-61.40） |
|  | TNF-α | 0.75  （0.67-0.83） | ＞0.28 | 71.43  （59.95-80.68） | 65.71  （54.04-75.75） |
|  | IL-4 | 0.81  （0.74-0.88） | ＞0.14 | 61.43  （49.72-71.95） | 100.00  （94.80-100.00） |
|  | IL-10 | 0.99  （0.99-1.00） | ＞4.76 | 100.00  （94.80-100.00） | 98.57  （92.34-99.93） |
|  | TGF-β | 0.99  （0.99-1.00） | ＞81.97 | 100.00  （94.80-100.00） | 98.57  （92.34-99.93） |
| **SNIs**  **vs**  **HDs** | IL-6 | 0.98  （0.96-0.99） | ＞0.95 | 100.00  （94.80-100.00） | 85.71  （75.66-92.05） |
|  | IL-1β | 0.98  （0.96-1.00） | ＞0.49 | 97.14  （90.17-99.49） | 80.00  （69.18-87.70） |
|  | TNF-α | 0.95  （0.92-0.98） | ＞0.90 | 91.43  （82.53-96.01） | 90.00  （80.77-95.07） |
|  | IL-4 | 0.60  （0.51-0.69） | ＞0.03 | 42.86  （31.94-54.52） | 72.86  （61.46-81.88） |
|  | IL-10 | 0.91  （0.87-0.96） | ＞1.02 | 100.00  （94.80-100.00） | 52.86  （41.32-64.10） |
|  | TGF-β | 0.76  （0.68-0.84） | ＞58.39 | 57.14  （45.48-68.06） | 85.71  （75.66-92.05） |

CI: confidence interval.

**Table S6.** Summary of median and range of clinically available indicators in the training cohort (n=210).

| Characteristic | SIs | SNIs | HDs | Total |
| --- | --- | --- | --- | --- |
| Total cases | 70 | 70 | 70 | 210 |
| CRP [mg L^-1^]  Median  Range | 69.33  10.12-212.55 | 98.94  11.02-237.77 | 3.38  0.09-87.67 | 57.21  0.09-237.77 |
| PCT [ng L^-1^]  Median  Range | 6.08  0.22-40.58 | 20.11  0.28-79.67 | 0.26  0.01-0.49 | 8.82  0.01-79.67 |
| TLC [×10^9^ L^-1^] |  |  |  |  |
| Median  Range | 0.92  0.12-4.54 | 1.14  0.08-8.76 | 2.15  1.03-3.26 | 1.41  0.08-8.76 |
| SA [g L^-1^]  Median  Range | 30.52  20.00-43.50 | 32.35  21.00-48.50 | 47.08  40.40-55.00 | 36.65  20.00-55.00 |

CRP: C-reactive protein; PCT: procalcitonin; TLC: total lymphocyte count; SA: serum albumin.

**Table S7.** AUCs, sensitivities and specificities of clinically available indicators for SIs and SNIs diagnosis in the training cohort (n = 210).

|  |  | **AUC**  **[95%CI]** | **Cutoff** | **Sensitivity [%]**  **[95%CI]** | **Specificity [%] [95%CI]** |
| --- | --- | --- | --- | --- | --- |
| **SIs**  **vs**  **SNIs** | CRP | 0.62  （0.53-0.72） | ＜102.40 | 80.00  （69.18-87.70） | 48.57  （37.25-60.05） |
|  | PCT | 0.71  （0.62-0.79） | ＜14.45 | 94.29  （86.21-97.76） | 44.29  （33.25-55.92） |
|  | TLC | 0.54  （0.44-0.63） | ＜0.87 | 65.71  （54.04-75.75） | 52.86  （41.32-64.10） |
|  | SA | 0.54  （0.44-0.64） | ＜30.15 | 57.14  （45.48-68.06） | 61.43  （49.72-71.95） |
| **SIs**  **vs**  **HDs** | CRP | 1.00  （1.00-1.00） | ＞8.90 | 100.00  （94.80-100.00） | 100.00  （94.80-100.00） |
|  | PCT | 0.97  （0.95-1.00） | ＞0.50 | 94.29  （86.21-97.76） | 100.00  （94.80-100.00） |
|  | TLC | 0.91  （0.86-0.97） | ＜1.14 | 80.00  （69.18-87.70） | 95.71  （88.14-98.83） |
|  | SA | 0.99  （0.99-1.00） | ＜39.50 | 97.14  （90.17-99.49） | 100.00  （94.80-100.00） |
| **SNIs**  **vs**  **HDs** | CRP | 1.00  （1.00-1.00） | ＞9.35 | 100.00  （94.80-100.00） | 100.00  （94.80-100.00） |
|  | PCT | 0.98  （0.96-1.00） | ＞0.51 | 97.14  （90.17-99.49） | 100.00  （94.80-100.00） |
|  | TLC | 0.88  （0.83-0.94） | ＜1.51 | 80.00  （69.18-87.70） | 81.43  （70.77-88.81） |
|  | SA | 0.92  （0.88-0.97） | ＜39.75 | 84.29  （74.01-90.99） | 100.00  （94.80-100.00） |

**Table S8.** Clinical characteristics of SIs, SNIs and healthy donors (60 samples for biomarker analysis, 300 independent samples for machine learning modeling).

| **Characteristic** | **SIs** | **SNIs** | **HDs** | **Total** |
| --- | --- | --- | --- | --- |
| **Total cases** | 120 | 120 | 120 | 360 |
| **Age [years]** |  |  |  |  |
| Median | 70 | 58 | 53 | 60 |
| Range | 33-89 | 31-87 | 19-89 | 19-89 |
| **Gender** |  |  |  |  |
| Male | 66 | 59 | 56 | 181 |
| Female | 54 | 61 | 64 | 179 |

**Table S9.** Multiclass diagnostic performance and overfitting-related indicators for machine learning models constructed with different Multi-Cytokines panels.

| **Model** | **Feature Set** | **Train**  **AUC** | **AUC (95%CI)** | **Sensitivity (95%CI)** | **Specificity (95%CI)** | **Accurac-y** | **CV AUC** | **AUC gap** | **Overfit**  **Flag** | **Log**  **Loss** | **Brier** |
| --- | --- | --- | --- | --- | --- | --- | --- | --- | --- | --- | --- |
| CatBoost | IL-1β+TNF-α+IL-10 | 0.999 | 0.999  (0.996-1.000) | 0.970  (0.934-1.000) | 0.985  (0.968-1.000) | 0.970 | 0.996 ± 0.004 | 0.001 | 0 | 0.129 | 0.018 |
| XGBoost | IL-1β+TNF-α+IL-10 | 1.000 | 0.998  (0.995-1.000) | 0.980  (0.949-1.000) | 0.990  (0.975-1.000) | 0.980 | 0.995 ± 0.004 | 0.001 | 0 | 0.110 | 0.017 |
| GBDT | IL-1β+TNF-α+IL-10 | 1.000 | 0.998  (0.995-1.000) | 0.970  (0.934-1.000) | 0.985  (0.969-1.000) | 0.970 | 0.994 ± 0.006 | 0.002 | 0 | 0.111 | 0.019 |
| SEL | IL-6+IL-1β+TNF-α+IL-10 | 1.000 | 0.999  (0.995-1.000) | 0.971  (0.933-1.000) | 0.985  (0.967-1.000) | 0.970 | 0.997 ± 0.002 | 0.001 | 0 | 0.096 | 0.015 |
| NB | IL-1β+TNF-α+IL-10+TGF-β | 0.999 | 0.994  (0.985-1.000) | 0.931  (0.884-0.972) | 0.965  (0.942-0.986) | 0.930 | 0.994 ± 0.008 | 0.005 | 0 | 0.243 | 0.039 |
| RNN | IL-1β+TNF-α+IL-4+TGF-β | 0.995 | 0.994  (0.983-0.999) | 0.851  (0.766-.910) | 0.925  (0.883-0.957) | 0.850 | 0.974 ± 0.009 | 0.001 | 0 | 0.699 | 0.131 |
| RF | IL-6+IL-1β+TNF-α+IL-10+TGF-β | 1.000 | 0.998  (0.993-1.000) | 0.970  (0.928-1.000) | 0.985  (0.964-1.000) | 0.970 | 0.998 ± 0.003 | 0.002 | 0 | 0.122 | 0.021 |
| RNT | IL-6+IL-1β+TNF-α+IL-4+TGF-β | 0.987 | 0.994  (0.987-0.999) | 0.910  (0.857-0.960) | 0.955  (0.931-0.980) | 0.910 | 0.985 ± 0.014 | -0.007 | 0 | 0.514 | 0.088 |
| MLP | IL-1β+TNF-α+IL-4+IL-10+TGF-β | 0.999 | 0.993  (0.983-0.999) | 0.930  (0.877-0.973) | 0.965  (0.936-0.986) | 0.930 | 0.998 ± 0.005 | 0.006 | 0 | 0.162 | 0.030 |
| CNN | IL-6+IL-1β+TNF-α+IL-4+IL-10+TGF-β | 0.998 | 0.996  (0.990-1.000) | 0.950  (0.908-0.989) | 0.975  (0.955-0.995) | 0.950 | 0.991 ± 0.004 | 0.002 | 0 | 0.286 | 0.045 |
| DNT | IL-6+IL-1β+TNF-α+IL-4+IL-10+TGF-β | 0.995 | 0.996  (0.989-1.000) | 0.920  (0.861-0.969) | 0.960  (0.932-0.984) | 0.920 | 0.987 ± 0.006 | -0.000 | 0 | 0.382 | 0.063 |
| GRU | IL-6+IL-1β+TNF-α+IL-4+IL-10+TGF-β | 0.990 | 0.990  (0.978-0.998) | 0.851  (0.775-.911) | 0.925  (0.888-0.955) | 0.850 | 0.845 ± 0.040 | 0.000 | 0 | 0.762 | 0.144 |

**References**

[1]	Cajander S., Kox M., Scicluna B., Weigand M., Mora R., Flohﺃﺏ S., et al. Profiling the dysregulated immune response in sepsis: overcoming challenges to achieve the goal of precision medicine. The Lancet Respiratory medicine 2024;12:305-22.

[2]	Paul D., Anower H., Daniel F M., David B., Gordon C., Jonathan C C., et al. Biomarker-Guided Antibiotic Duration for Hospitalized Patients With Suspected Sepsis: The ADAPT-Sepsis Randomized Clinical Trial. JAMA 2024;333.

[3]	L Nelson S.-P., Tellen D B., Peter E D., Seth R., Margaret N R., Blake M., et al. Development and Validation of the Phoenix Criteria for Pediatric Sepsis and Septic Shock. JAMA 2024;331.

[4]	Erin F C., Mallory A P.-E., Hallie C P. Context and Implications of the New Pediatric Sepsis Criteria. JAMA 2024;331.

[5]	Luregn J S., R Scott W., Lauren R S., Andrew C A., Kusum M., Mark W H., et al. International Consensus Criteria for Pediatric Sepsis and Septic Shock. JAMA 2024;331.

[6]	van der Poll T., van de Veerdonk F. L., Scicluna B. P., Netea M. G. The immunopathology of sepsis and potential therapeutic targets. Nature Reviews Immunology 2017;17:407-20.

[7]	Delano M. J., Ward P. A. The immune system's role in sepsis progression, resolution, and long-term outcome. Immunological Reviews 2016;274:330-53.

[8]	Li H., Jiang S., Chen Z., Yao Y., Chen M., Hu Y. Identification and validation of core biomarkers for sepsis: a comprehensive analysis using bioinformatics and machine learning. Frontiers in Immunology 2025;16.

[9]	Boomer J. S., To K., Chang K. C., Takasu O., Osborne D. F., Walton A. H., et al. Immunosuppression in Patients Who Die of Sepsis and Multiple Organ Failure. JAMA 2011;306:2594-605.

[10]	Zheng L.-y., Duan Y., He P.-y., Wu M.-y., Wei S.-t., Du X.-h., et al. Dysregulated dendritic cells in sepsis: functional impairment and regulated cell death. Cellular & Molecular Biology Letters 2024;29.

[11]	Shao R., Fang Y., Yu H., Zhao L., Jiang Z., Li C.-S. Monocyte programmed death ligand-1 expression after 3–4 days of sepsis is associated with risk stratification and mortality in septic patients: a prospective cohort study. Critical Care 2016;20.

[12]	Torres L. K., Pickkers P., van der Poll T. Sepsis-Induced Immunosuppression. Annu Rev Physiol 2022;84:157-81.

[13]	Evans L., Rhodes A., Alhazzani W., Antonelli M., Coopersmith C. M., French C., et al. Surviving sepsis campaign: international guidelines for management of sepsis and septic shock 2021. Intensive Care Medicine 2021;47:1181-247.

[14]	Prescott H. C., Antonelli M., Alhazzani W., Møller M. H., Alshamsi F., Azevedo L. C. P., et al. Surviving Sepsis Campaign: International Guidelines for Management of Sepsis and Septic Shock 2026. Critical Care Medicine 2026.

[15]	Shankar-Hari M., Phillips G. S., Levy M. L., Seymour C. W., Liu V. X., Deutschman C. S., et al. Developing a New Definition and Assessing New Clinical Criteria for Septic Shock: For the Third International Consensus Definitions for Sepsis and Septic Shock (Sepsis-3). JAMA 2016;315:775-87.

[16]	Hegde S., Leader A. M., Merad M. MDSC: Markers, development, states, and unaddressed complexity. Immunity 2021;54:875-84.

[17]	Monneret G., Lafon T., Gossez M., Evrard B., Bodinier M., Rimmelé T., et al. Monocyte HLA-DR expression in septic shock patients: insights from a 20-year real-world cohort of 1023 cases. Intensive Care Medicine 2025;51:1820-32.

[18]	Cheng L., Li Y., Ji W., Yan J. An integrated immunofluorescent detection system for automated and sensitive protein quantification based on a microfluidic flow cytometry platform. Anal Chim Acta 2025;1341:343658.

[19]	Amid S., Lubna N., Shadman K., Lei T., Liane L., Hareet S., et al. Noncontact 3D Bioprinting of Proteinaceous Microarrays for Highly Sensitive Immunofluorescence Detection within Clinical Samples. ACS Nano 2024;18.

[20]	Soto D., Serafﺃﺝn V., Pedrero M., Pingarrﺃ٣n J. M., Campuzano S., Orozco J. Hierarchical Au@Pt nanoparticle/amino benzoic acid polymer-based hybrid material for labeled and label-free detection of interleukin-6: a comparative assessment. Mikrochim Acta 2024;191:683.

[21]	Li T., Kosgei B. K., Soko G. F., Meena S. S., Cao Q., Hou X., et al. An immunosensor for the near real-time and site of inflammation detections of multiple proinflammatory cytokines. Biosens Bioelectron 2024;263:116618.

[22]	Huiyu Z., Wuming W., Jingjing Z., Chunyan D. SILAR Growth of ZnO NSs/CdS QDs on the Optical Fiber-Based Opto-Electrode with Guided In Situ Light and Its Application for the "Signal-On" Detection of Inflammatory Cytokine. Anal Chem 2024;96.

[23]	Ao L., Xiufang M., Yu L., Geng Z., Chang L., Xi Y., et al. Digital SERS immunoassay of Interleukin-6 based on Au@Ag-Au nanotags. Biosens Bioelectron 2024;270.
